# WNT signaling coordinately controls mouse limb bud outgrowth and establishment of the digit-interdigit pattern

**DOI:** 10.1242/dev.204606

**Published:** 2025-06-10

**Authors:** Jonas Malkmus, Angela Morabito, Lucille Lopez-Delisle, Laura Avino-Esteban, Alexandre Mayran, Aimee Zuniga, James Sharpe, Rolf Zeller, Rushikesh Sheth

**Affiliations:** ^1^ Developmental Genetics, Department of Biomedicine, University of Basel, CH4054 Basel, Switzerland; ^2^ School of Life Sciences, École Polytechnique Fédérale de Lausanne (EPFL), CH 1015 Lausanne, Switzerland; ^3^ Multicellular Systems Biology, European Molecular Biology Laboratory (EMBL-Barcelona), Barcelona 08003, Spain

**Keywords:** Digit-interdigit pattern, Limb bud development, Plasticity, Robustness, Self-regulation, Signaling system, WNT/β-catenin

## Abstract

Self-organization, such as the emergence of a pattern from a homogenous state, is a fascinating property of biological systems. Early limb bud outgrowth and patterning in mice are controlled by a robust and self-regulatory signaling system, and initiation of the periodic digit-interdigit pattern appears to be under the control of a self-regulatory Turing system. Previous studies established the requirement of WNT and BMP signaling for both early limb bud and digit-interdigit morphogenesis, but the molecular changes underlying the transition from early limb bud signaling to the digit-interdigit patterning system remained unknown. Here, we have used small molecule inhibitors to rapidly but transiently block WNT signaling to identify the early transcriptional targets that are altered during disruption and recovery of limb bud and digit development. Together, this study highlights the overarching role of WNT signaling in controlling early limb bud outgrowth and patterning, and the establishment of the periodic digit-interdigit pattern. Finally, the transient WNT signaling disruption approach reveals the plasticity and robustness of these self-organizing limb bud- and digit-patterning systems.

## Teaser

**Highlighted Article:** Small molecule inhibitor studies reveal for the first time that WNT signaling controls the transition of early limb bud development to the periodic digit-interdigit patterning system.

## INTRODUCTION

**Fig. 1.**
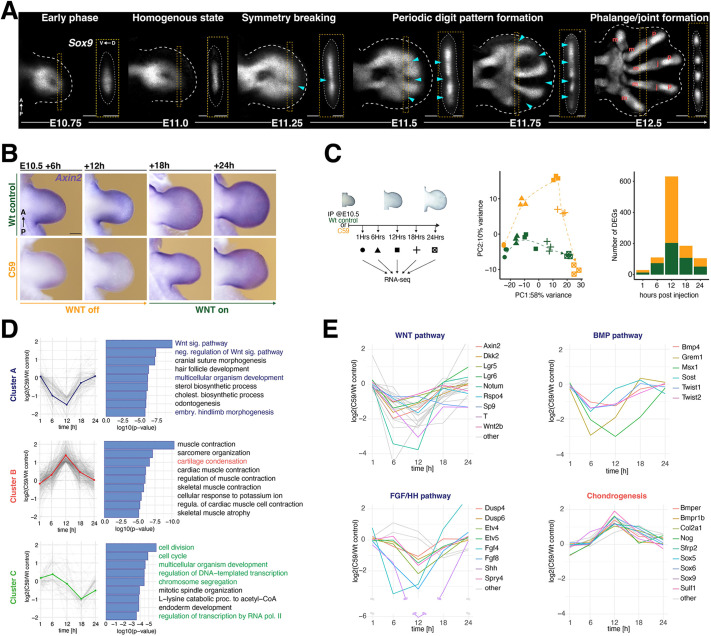
**C59-mediated inhibition of WNT signaling is transient and temporarily deregulates limb bud transcriptional programs.** (A) Whole-mount RNA-FISH in wild-type forelimb buds between E10.75 and E12.5 shows the spatial distribution of *Sox9* expression from early limb bud outgrowth (E10.75) to late stage digit and phalange development. The formation of the first interdigit at symmetry breaking and the progressive establishment of periodic digit/metacarpal interdigit pattern is shown (blue arrowheads indicate interdigits). By E12.5, digits 4-2 are composed of metacarpals (m), joints (j) and phalanges (p). A, anterior; P, posterior; D, dorsal; V, ventral. Scale bars: 200 µm. (B) *Axin2* expression in wild-type control (DMSO-treated) and C59-treated forelimb buds at ∼E10.5+6, +12, +18 and +24 h after injection. *n*=3 independent biological replicates per stage. Scale bar: 250 µm. (C) At the specific timepoints indicated, wild-type control and C59-treated forelimb buds from embryos were processed for total RNA-sequencing (scheme on the left, *n*=3 independent biological replicates). PCA analysis shows the differences between wild-type and C59-treated samples at the different timepoints (middle panel). The bar plots on the right represent numbers of DEGs for each timepoint. Green, wild-type control; orange, C59-treated samples. (D) DEGs were split into three clusters (A-C) using their profile of fold-changes over time (Fig. S5). For each DEG cluster, GO analysis identified the top 10 enriched biological processes. GO processes most relevant for this study are in colored text. (E) Selected DEGs associated with the WNT, BMP and FGF/HH pathways for cluster A (highlighted in blue in D) are shown as line plots. Line plots are also shown for selected DEGs in cluster B that are linked to chondrogenic processes (highlighted in red in D). For the FGF/HH pathway, an axis break in the −log2 scale and *Shh* line plot is indicated.

The developing limb bud is a paradigm with which to study the interplay between growth, patterning and tissue morphogenesis. Briefly, WNT, BMP and FGF signaling are first required to establish the dorsal-ventral (DV) limb bud axis and apical ectodermal ridge (AER) during limb bud formation (Geetha-Loganathan et al., 2008; Mariani et al., 2008; Soshnikova et al., 2003; Zeller et al., 2009). In turn, AER-FGF signaling is essential to promote proximal-distal (PD) outgrowth, while the posterior sonic hedgehog (SHH) signaling center is required for anterior-posterior (AP) limb bud patterning (Zuniga and Zeller, 2020). Transcriptional activation of the BMP antagonist gremlin 1 (*Grem1*) by BMP and SHH signaling results in establishment of the robust and self-regulatory SHH/GREM1/AER-FGF signaling system (Bénazet et al., 2009; Zuniga et al., 1999). This signaling system promotes survival and expansion of limb bud mesenchymal progenitors (LMPs), including the progenitors that will give rise to the autopod primordia and digits (Markman et al., 2023; Michos et al., 2004; Palacio et al., 2025). Around embryonic day E11.25, a gap appears in the distal crescent of the *Sox9* expression domain in mouse forelimb buds, which is the earliest sign of breaking the AP symmetry of the *Sox9* domain (Fig. 1A). This marks the onset of establishing the periodic digit-interdigit pattern as the *Sox9*-expressing cells will give rise to digits, while cells that cease expression become interdigital or soft tissue (Fig. 1A, Akiyama et al., 2005; Bénazet et al., 2012; Raspopovic et al., 2014). Subsequently (∼E11.75), organizing centers termed phalange-forming regions (PFRs) are established at the distal tip of the metacarpal primordia to control distal growth and extension of digits, and phalange formation (Fig. 1A, Fig. S1, Grall et al., 2024; Huang and Mackem, 2022; Montero et al., 2008; Suzuki et al., 2008). While the molecular interactions and gene networks controlling these major morphogenetic processes are well studied, the interactions controlling the transition from early limb bud outgrowth and patterning to periodic digit-interdigit patterning are unknown.

Two morphogen-based hypotheses, positional information and self-organizing reaction-diffusion (RD), have been proposed (Green and Sharpe, 2015; Kondo and Miura, 2010): the positional information hypothesis provides a framework to study specification of AP positional identities in the limb bud mesenchyme by a morphogen gradient, which ultimately endows digits with distinct AP identities (Wolpert, 1969). SHH was identified as the proposed morphogen (Harfe et al., 2004; Riddle et al., 1993), but recent genetic and molecular analysis shows that rather than acting long-range, SHH establishes posterior identities already during onset of limb bud development (Zhu et al., 2008, 2022). However, the positional information model and the early specification of AP positional identities cannot explain the periodic digit-interdigit pattern (Sheth et al., 2012). With regard to results from data-based simulation, it has been proposed that digit-interdigit patterning is controlled by a self-organizing RD Turing system (Newman and Frisch, 1979; Raspopovic et al., 2014; Sheth et al., 2012). These simulation led to the proposal that the periodic pattern emerges as a consequence of local interactions among reacting and diffusing proteins, in *sensu stricto* not depending on positional information. From other Turing patterning systems, it is known that interactions of at least two morphogens with different diffusion rates disrupt the homogenous state and produce spatially opposing patterns, which manifest themselves by repeating spots or stripes (Gierer and Meinhardt, 1972; Kondo and Miura, 2010; Turing, 1952). Several studies provide insight and support for self-organizing Turing mechanisms acting during distal limb bud patterning. Molecular analysis supports the involvement of Turing-type systems in the initial periodic AP patterning of metacarpals (Raspopovic et al., 2014; Sheth et al., 2012), subsequent reiterative joint formation during digit primordia outgrowth (Grall et al., 2024) and emergence of fingerprint patterns (Glover et al., 2023).

WNT/β-catenin signaling plays an essential role from the initiation of limb bud outgrowth to the establishment of the periodic digit-interdigit pattern. Previous studies have shown that, in early limb buds, ectodermal WNT and AER-FGF maintain the underlying LMPs in an proliferative state and suppress chondrogenesis by restricting *Sox9* and BMP activity to the core mesenchyme (Berge et al., 2008; Reinhardt et al., 2019). With respect to digit-interdigit patterning, a Turing model derived from mathematical simulations of experimental data incorporating WNT and BMP signaling, and the transcription factor SOX9 predicts that interdigit BMP signaling promotes *Sox9* expression in digit primordia, while SOX9 restricts *Bmp2* expression to the interdigit (Raspopovic et al., 2014). In contrast, WNT signaling and SOX9 are mutually antagonistic, such that WNT inhibits *Sox9* expression in the interdigit, while SOX9 represses *Wnt* from digit territories. This BMP-SOX9-WNT (BSW) Turing network provides useful predictions for testing the molecular interactions that control the periodic digit-interdigit pattern.

Here, we initially used conditional inactivation of β-catenin (*Ctnnb1*) in mouse embryos, which supports the requirement of canonical WNT signaling in establishing the digit-interdigit pattern. However, genetic analysis is limited by incomplete conditional inactivation and embryonic lethality, which hinders analysis of digit development. To overcome these limitations, we resorted to intraperitoneal (IP) injection of the small molecule inhibitor Wnt-C59, which induces fast but transient disruption of WNT signaling. This approach allowed the identification of immediate transcriptional targets of WNT/β-Catenin in early mouse forelimb buds (∼E10.5). Molecular and cellular analysis reveals the positive impact of WNT signaling on transcriptional targets in the SHH/GREM1/AER-FGF signaling system and on limb bud cell proliferation. In addition, this analysis provided insight into the transition from this early limb bud signaling system to periodic digit-interdigit patterning. WNT inhibition disrupts initial establishment of the periodic digit-interdigit pattern, while the expression of genes functioning in chondrogenesis and digit patterning, such as *Sox9*, *Sfrp2* and *Sulf1*, are expanded. During progressive restoration of WNT signaling, the periodic digit-interdigit pattern recovers. Gene expression profiling identifies *Wnt2* and *Wnt2b* as ligands expressed by the interdigit mesenchyme, and *Sfrp2* and *Sulf1* as candidate signaling modulators that could restrict WNT/β-catenin signaling to the interdigit mesenchyme. Finally, this analysis provides evidence that *Sfrp2* and *Sulf1* could function as part of the Turing-type periodic digit-interdigit patterning system.

## RESULTS

### Conditional inactivation of β-catenin reveals the genetic requirement of canonical WNT signaling for digit patterning

The requirement of canonical WNT/β-catenin signaling for limb bud development has previously been studied using two different genetic approaches. Initially, a tamoxifen-inducible pan-active CRE-transgene (Hayashi and McMahon, 2002) was used for conditional inactivation of β-catenin (Huelsken et al., 2001) in early limb buds (∼E10.0, *Ctnnb1*^Δc/Δc^, Fig. S2). The expression of *Axin2*, a direct transcriptional target of canonical WNT signaling, is reduced but not completely lost after 48 h (Fig. S2A), together with genes functioning in early limb bud outgrowth and patterning (Fig. S2B,C; Hill et al., 2006; Jho et al., 2002). In addition, *Sox9* expression expands into the sub-ectodermal mesenchyme and the presumptive digit territory in *Ctnnb1*^Δc/Δc^ forelimb buds, which disrupts establishment of the periodic *Sox9* pattern marking digit primordia at embryonic day E12.0 (Fig. S2D). The lethality of *Ctnnb1*^Δc/Δc^ embryos (≤E13.5) precluded analysis of limb skeletal development. In contrast, *Hoxa13*-CRE-mediated conditional β-catenin inactivation causes loss of *Axin2* from the presumptive digit territory (Fig. S2E). The loss of the periodic *Sox9* expression, accompanied by its expansion into the distal-most mesenchyme (Fig. S2F), causes metacarpal fusions and loss of phalanges (Fig. S2G, Uenal, 2015). This genetic analysis shows that canonical WNT/β-catenin signaling is required for both the initial digit-interdigit and subsequent phalange pattern. However, the slow and incomplete inactivation (Fig. S2A) does not allow discrimination of specific WNT functions in early limb buds and subsequent digit-interdigit patterning.

### Transcriptional alterations in response to disrupting WNT signaling in mouse limb buds

To overcome the limitations of genetic inactivation, we evaluated the efficacy of three different small molecule inhibitors, IWP2 (Chen et al., 2009), ETC-159 (Madan et al., 2016) and C59 (Proffitt et al., 2013), in blocking WNT signaling. In pilot experiments, each component was injected intraperitoneally into pregnant mice at gestational day ∼E10.5 (Fig. S3). This analysis showed that IWP2 only partially inhibits WNT activity, as *Axin2* expression remains (Fig. S3A). In contrast, ETC-159 and Wnt-C59 significantly reduced *Axin2* expression within 6 h (Fig. S3A). Wnt-C59 was selected because it caused forelimb oligodactylies (three to four digits, Fig. S3C,D) with high penetrance, which was not the case for ETC-159 (Fig. S3A,B). C59 inhibits porcupine activity, an enzyme required for WNT ligand secretion, thereby blocking both canonical and non-canonical signaling (Proffitt et al., 2013). Of additional relevance to the chosen approach, Wnt-C59 has been successfully used to block WNT signaling during mouse embryonic and axolotl limb bud development (Lovely et al., 2022; Szenker-Ravi et al., 2018). As the periodic digit pattern is disrupted in most C59-treated hindlimbs (Fig. S4), the analysis focused on forelimb bud development. Indeed, a single IP injection of C59 (10 µg/g body weight) at E10.5 causes rapid reduction of *Axin2* expression (Fig. 1B). From 6 to 12 h, *Axin2* expression is reduced to low levels, but recovers within ∼18 to 24 h, revealing the rapid but transient nature of WNT signaling disruption in mouse limb buds (right panels, Fig. 1B).

To investigate the impact on gene expression, forelimb buds of DMSO solvent-injected, i.e. wild-type control and C59-treated mouse embryos, were collected for RNA-sequencing (RNA-seq) 1 h after injection at ∼E10.5, during disruption of WNT signaling (at +6 and +12 h) and recovery (at +18 and +24 h; left panel in Fig. 1C, Table S1). PCA analysis shows the distinct temporal trajectories of the transcription profiles following inhibition of WNT signaling in wild-type control and C59-treated forelimb buds (middle panel, Fig. 1C). In both cases, the gene expression profiles separate along the PC1 axis according to developmental time. Along the PC2 axis, the C59-treated forelimb bud samples diverge from their wild-type controls between +6 and +18 h (middle panel, Fig. 1C). This points to transient transcriptional differences in the gene expression profiles of C59-treated limb buds, which is corroborated by an increase in differentially expressed genes (DEGs) that peaks at +12 h post-C59 injection (right panel, Fig. 1C). Subsequently, the transcriptional differences are reduced and restored close to wild-type levels in C59-treated forelimb buds by +24 h (middle and right panel, Fig. 1C). Clustering of DEGs using their profiles of fold changes over time identifies three gene clusters with distinct transcriptional kinetics (Fig. 1D, Fig. S5). In cluster A, DEGs are rapidly downregulated, reaching maximum reductions between 6 and 12 h, followed by restoration close to wild-type levels by +24 h. Many DEGs in cluster A function as part of the major signaling pathways required for mouse limb bud outgrowth and patterning (Fig. 1E, Table S2), which includes the WNT, BMP, FGF and SHH pathways (Fig. 1E; Table S3). Conversely, cluster B is enriched in genes that function in chondrogenesis and myogenesis (Fig. 1D, Table S1). In particular, DEGs that function in chondrogenesis and/or digit-interdigit patterning, such as *Sox9*, *Bmpr1b*, *Sfrp2* and *Sulf1* (Figs 5 and 6), are precociously upregulated after IP injection of Wnt-C59. This upregulation is maximal at +12 h and reduced again to close to wild-type levels by +24 h (Fig. 1E, Fig. S4B, Table S2). Cluster C is enriched in DEGs that function in regulation of the cell cycle and cell division (Fig. 1D, Table S1). The delayed transcriptional downregulation of DEGs in cluster C peaks at +18 h and is only partially restored at +24 h (Fig. 1D, see also below).

### Identification of the early transcriptional targets of WNT/β-catenin signaling in mouse limb buds

**Fig. 2.**
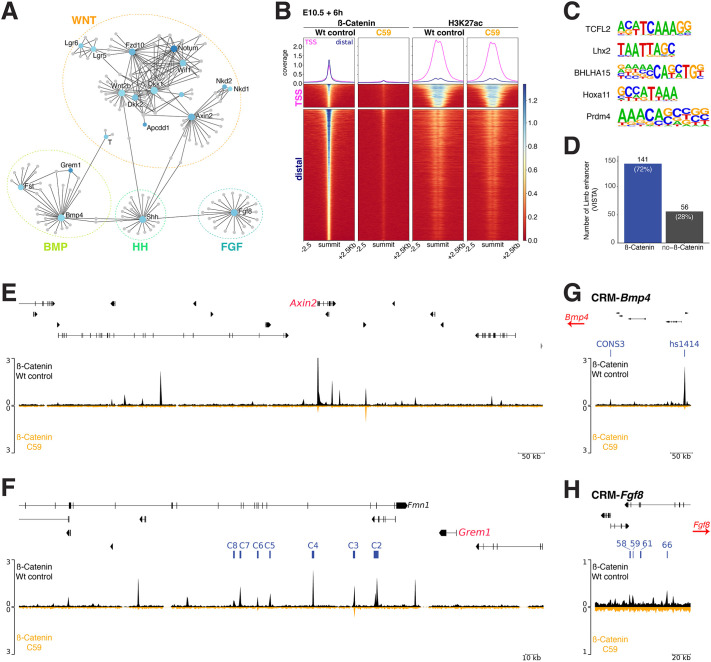
**Identification of WNT-dependent gene regulatory networks and *cis-*regulatory landscapes in forelimb buds.** (A) Genes downregulated at +6 h post C59 treatment were used to generate curated gene interaction networks. Subnetwork 1 with 24 seeds (genes) is shown after removing the *Igf1*, *Bdnf*, *Ngf*, *Tac1* and *Ccl28* seeds. (B) β-Catenin and histone H3K27Ac ChIP-seq analysis reveals the interactions with chromatin and the modification profiles, respectively, in wild-type control and C59-treated forelimb buds at E10.5+6 h (*n*=2 biological replicates). Line plots show the β-catenin distribution and H3K27ac profiles at transcriptional start sites (TSS) and distal regions. Heatmaps show the presence or absence of β-catenin interactions and overlapping H3K27ac modifications in wild-type control and C59-treated limb buds. (C) The top five *de novo* consensus motifs enriched in the β-catenin ChIP-seq profile. (D) Intersection of β-catenin ChIP-seq peaks with enhancers active in mouse limb buds. 141 out of 197 VISTA limb bud enhancers are enriched in β-catenin chromatin complexes. (E) *Axin2* genomic region (exons shown as black bars above), the β-catenin ChIP-seq profile (E10.5+6 h) is plotted for wild-type (black profile) and C59-treated limb buds (orange profile). (F) The *Grem1 cis*-regulatory landscape encodes seven CRMs (C2-C8, indicated in blue) that are required for *Grem1* expression in mouse limb buds (exons indicated above). All seven CRMs are enriched in β-catenin chromatin complexes (black profile). These ChIP-seq peaks are reduced or lost after C59 treatment (orange profile). (G) CRM-*Bmp4*: CONS3 is a validated enhancer regulating *Bmp4* expression; VISTA hs1414 is an apical ectodermal ridge (AER)-specific enhancer. Both CRMs (blue) are enriched in β-catenin ChIP-seq peaks (black profile), which are lost after C59 treatment. (H) CRM-*Fgf8*: four CRMs in the *Fgf8* genomic landscape are active in the AER (blue). Three of them are marked by β-catenin ChIP-seq peaks (black profile), which are lost after C59 treatment (orange profile).

C59-mediated WNT inhibition shows that genes in the BMP, FGF and SHH signaling pathways are downregulated with similar rapid kinetics (≤6 h, Fig. 1E), indicating that they are early targets of WNT signal transduction. Therefore, network analysis using these DEGs was performed to identify the gene regulatory networks (GRNs) whose expression is positively regulated by WNT signaling (Fig. 2A, Table S4). This GRN analysis pinpoints the likely primary targets in the different signaling pathways. To determine if these alterations could depend on direct regulation by WNT/β-catenin, specific antibodies for β-catenin and H3K27ac, which marks active chromatin regions (Andrey et al., 2017), were used for chromatin immunoprecipitation sequencing (ChIP-seq) of wild-type control and C59-treated limb buds at E10.5+6 h (∼E10.75, Fig. 2B). The interaction of β-catenin with regions in proximity to transcriptional start sites (TSSs) and distal regions in wild-type limb buds establishes that β-catenin predominantly interacts with distal *cis*-regulatory modules (CRMs; Fig. 2B). Motif analysis identifies a TCF/LEF binding sequence as the top enriched motif (Fig. 2C). This is expected from β-catenin-forming complexes with TCF/LEF1 transcriptional regulators in mediating response to canonical WNT signal transduction (Cadigan and Waterman, 2012; Söderholm and Cantù, 2021). Within 6 h of C59-treatment, β-catenin binding is depleted at TSS and distal regions in comparison with wild-type limb buds (Fig. 2B). In contrast, there are no obvious changes in the H3K27ac profiles by comparing C59-treated to wild-type limb buds at +6 h (Fig. 2B). Furthermore, 141 of 197 (72%) of validated VISTA enhancers active in limb buds overlap regions enriched in β-catenin complexes (Fig. 2D, Kosicki et al., 2024; Visel et al., 2007). Next, the β-catenin interactions with *cis*-regulatory regions and the H3K27 acetylation patterns in the genomic landscapes of selected DEGs at E10.5+6 h were comparatively analyzed (Fig. 2E-H). In the *Axin2* genomic landscape, β-catenin peaks in regions that could mediate the rapid feedback between WNT/β-catenin signal transduction and *Axin2* transcription were identified in wild-type forelimb buds (Fig. 2E). In agreement with the loss of *Axin2* expression following C59 treatment (Fig. 1B), β-Catenin is depleted from CRMs at E10.5+6 h (Fig. 2E). As the RNAseq analysis indicated, the BMP antagonist *Grem1* and *Bmp4* ligand are early and positively regulated WNT target genes (Figs 1D and 2A); therefore, the interaction of β-catenin with both loci was assessed (Fig. 2F,G). Indeed, both the limb enhancers in the *Grem1* genomic landscape (Malkmus et al., 2021) and an enhancer regulating *Bmp4* expression (Jumlongras et al., 2012) are enriched in β-catenin complexes in wild-type limb buds, while these interactions are lost within 6 h of C59 treatment (Fig. 2F,G). This is also the case for three enhancers regulating *Fgf8* expression in the limb bud AER (Fig. 2H, Marinić et al., 2013) and for the β-catenin peak at the ZRS enhancer controlling *Shh* expression in limb buds (Fig. S6E, Lettice et al., 2003). In contrast, only minor alterations are apparent in the H3K27ac patterns in these genomic landscapes upon blocking WNT/β-Catenin signaling (E10.5+6 h, Fig. S6).

### WNT signaling is required for the self-regulatory signaling system that controls limb bud outgrowth and patterning

**Fig. 3.**
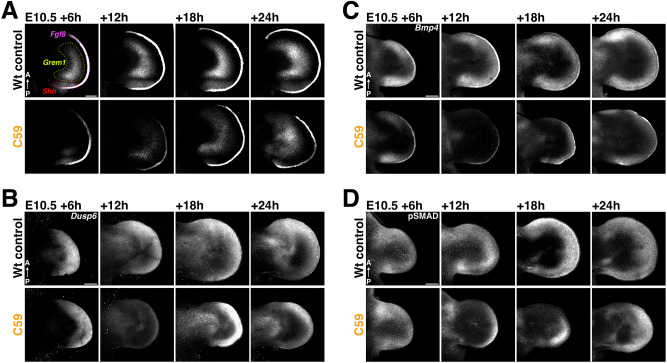
**The spatio-temporal response of the self-regulatory SHH/GREM1/AER-FGF signaling system to inhibition of WNT signaling.** (A-C) RNA-FISH analysis at E10.5+6, +12, +18 and +24 h in wild-type control and C59-treated limb buds. For all limb buds, the entire *z*-stack is shown as a maximum intensity projection. (A) *Shh*, *Grem1* and AER-*Fgf8* expression assessed in the same forelimb buds using the same fluorophore for all three probes. The *Shh* expression domain is marked in red, *Grem1* in green and *Fgf8* in the AER in magenta. (B) Expression of the FGF target gene *Dusp6*. (C) Spatial dynamics of *Bmp4* expression in wild-type and C59-treated forelimb buds. (D) Whole-mount immunostaining of the pSMAD (pSMAD1,5,9) distribution reveals the spatial distribution of active BMP signal transduction. A selected *z*-stack range (midsection) is shown as maximum intensity projections. *n*=3 biological replicates were analyzed for all time points shown in A-D. A, anterior; P, posterior. Scale bars: 200 µm.

The analysis thus far establishes that WNT signaling positively regulates genes functioning in the self-regulatory limb bud signaling system (Fig. 2, Fig. S6), which interlinks mesenchymal SHH with BMP4/GREM1 and AER-FGF signaling to coordinate AP axis pattering with PD limb bud outgrowth. To assess the impact of transient WNT signal disruption on spatial gene expression, *Shh*, *Grem1* and *Fgf8* HCR probes were multiplexed for fluorescent whole-mount RNA *in situ* hybridization (RNA-FISH, Fig. 3A). Analyzing their expression in the same forelimb buds reveals the rapid reduction of *Grem1* transcripts and AER-*Fgf8* expression (at +6 to +12 h), which is followed by progressive recovery after +18 to +24 h (∼E11.0-E11.5, Fig. 3A). This downregulation is paralleled by almost complete loss of AER-FGF signal transduction, as reflected by the reduced expression of the FGF transcriptional target *Dusp6* in the distal limb bud mesenchyme (∼E11.0-E11.5, Fig. 3B, Kawakami et al., 2003). The transient disruption not only lowers *Grem1* transcripts (Fig. 3A) and protein (Fig. S7A) but also mesenchymal *Bmp4* expression (Fig. 3C). The reduction and recovery of BMP activity is apparent from the spatio-temporal kinetics of SMAD activities (Fig. 3D) and the expression changes of BMP target genes in C59-treated limb buds (*Msx2* and *Id1*; Fig. S7B,C). Posterior *Shh* expression is reduced but recovers to a lesser extent (Fig. 3A, see also Fig. S7E), which is reflected by reduced expression of its transcriptional target *Ptch1* (Fig. S7D). Following transient WNT inhibition, the limb bud signaling system scales down by +6 h, but largely recovers by +24 h (Fig. 3).

In comparison to wild-type controls, autopod primordia of C59-treated embryos are consistently smaller at E10.5+18, +24 h (∼E11.5; Fig. 3, Fig. S7A-E). Therefore, we assessed if inhibiting WNT signaling affects cell survival and proliferation (Fig. S7F,G). Comparative analysis of cleaved caspase 3 in wild-type and C59-treated forelimb buds detected localized and transient apoptosis at +6 and +12 h in the anterior-proximal part of the developing autopod (Fig. S7F). However, no apoptosis is detected in the distal limb bud mesenchyme, which harbors the LMP populations that will give rise to digits (Palacio et al., 2025). In addition, the cell cycle was analyzed by flow cytometry, which established that cell proliferation starts to decrease at +6 h (S phase) and is significantly reduced by +12 and +18 h after C59 treatment (Fig. S7G, left-most panel). In particular, the fraction of cells in G1 is increased, while the fraction of cells in S (both timepoints) and G2/M phase (+18 h) are decreased in comparison to wild-type controls (Fig. S7G). At +24 h, proliferation in C59-treated limb buds is increased again and levels are higher than in wild-type controls (left- and right-most panel, Fig. S7G). This disparity is a likely consequence of the decrease in wild-type LMP proliferation rates by E11.5-E11.75 (Reinhardt et al., 2019). This transient alteration in cell proliferation is in line with the WNT requirement for cell proliferation that was uncovered by the initial DEG analysis (see cluster C in Fig. 1D). This analysis establishes WNT signaling as an integral part of the self-regulatory signaling system that regulates the proliferative expansion of the LMP populations that will give rise to the autopod primordia and digits (Desanlis et al., 2020; Markman et al., 2023; Palacio et al., 2025; Sheth et al., 2013).

### WNT inhibition during early limb bud outgrowth also disrupts subsequent digit-interdigit patterning

**Fig. 4.**
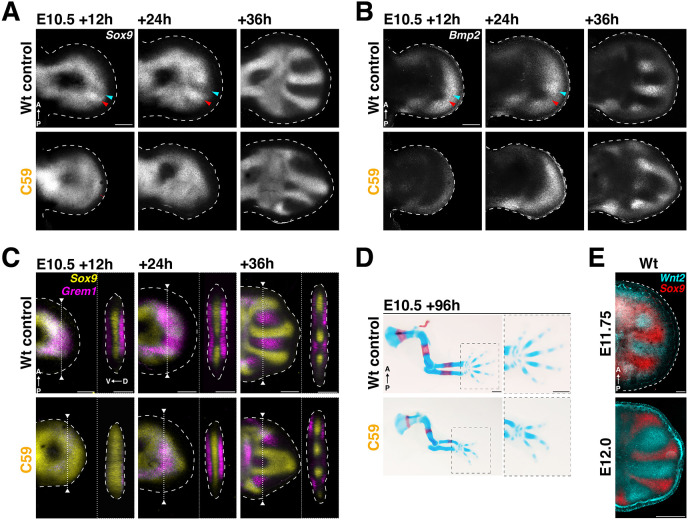
**Destabilization and re-establishment of the digit pattern during transient disruption of WNT signaling.** RNA-FISH analysis of gene expression dynamics in wild-type control and C59-treated forelimb buds (∼E10.5). The timepoints shown cover the disruption (+12 h) and recovery of WNT signaling (+24, +36 h). (A) *Sox9* expression dynamics. (B) *Bmp2* expression dynamics. (C) *Sox9* (yellow) and *Grem1* (magenta) expression dynamics. Right panels show virtual cross-sections at the level indicated by dotted lines in the left panels. (D) Alizarin Red (bone)/Alcian Blue (cartilage) staining to analyze the limb skeleton of wild-type control and C59-treated forelimb buds analyzed at E10.5+96 h. Disrupting WNT signaling induces oligodactylies: 4 digits (39%), 3 digits (52%), 2 digits (6%) and 1 digit (3%). *n*=31 biological replicates. (E) Co-detection of *Sox9* (red) and *Wnt2* (cyan) by RNA-FISH. (A-C,E) Selected z-stack ranges are shown as maximum intensity projections. Scale bars: 500 µm in D; 200 µm in A-C,E.

C59-mediated transient disruption of WNT signaling at E10.5 also allows the assessment of the effects of WNT signaling on digit ray patterning, as the progressive recovery of WNT signaling (Fig. 1B-E) overlaps the time window of symmetry breaking (∼E11.25, Fig. 1A) and the establishment of the periodic digit-interdigit pattern (≥E11.5, Fig. 1A). To gain insight into the effects on pattern formation, *Sox9* expression was analyzed in wild-type control and C59-treated limb buds between 12 h and 36 h after injection (∼E10.5, Fig. 4A). In contrast to the appearance of the first digit-interdigit separation in wild-type forelimb buds (red and blue arrowheads in Fig. 4A), the *Sox9* expression domain is distally expanded and there is no sign of ongoing digit-interdigit separation after C59 treatment (lower left panel in Fig. 4A). This is consistent with WNT/β-catenin signaling restricting *Sox9* expression likely by binding to CRMs in its genomic landscape (VISTA enhancers, Fig. S8A). During recovery of WNT signaling (Fig. 1B,C), *Sox9* expression is restricted from the distal-most mesenchyme and a periodic digit-interdigit pattern is established by E10.5+36 h (∼E11.5-E12.0, lower middle and right panels in Fig. 4A). The temporal expression dynamics of BMP receptor 1b (*Bmpr1b*) are similar to *Sox*9 during distal progression of limb bud development (upper panels in Fig. S9A, see also Fig. 1E). After C59 treatment, *Bmpr1b* expression also expands into the distal-most mesenchyme at E10.5+12 h (∼E11.0, lower left panel in Fig. S9A). During recovery of WNT signaling, *Bmpr1b* expression is progressively re-restricted to the forming digit primordia by +36 h (∼E11.5-E12.0, lower middle and right panels in Fig. S9A). Conversely, *Bmp2* is distally expressed and restricts to the interdigit mesenchyme during digit-interdigit patterning (Fig. 4B), while its expression declines during WNT signaling disruption (Fig. 4B), consistent with loss of β-catenin-CRM interactions (Fig. S8B, Dathe et al., 2009). During recovery of WNT signaling, *Bmp2* expression is progressively re-established and restricted to the interdigit region at +36 h (∼E11.5-12.0, lower right panel in Fig. 4B). In addition to *Bmp2*, the crescent-shaped *Grem1* expression domain in the dorsal and ventral mesenchyme (upper left panel in Fig. 4C) becomes restricted to the forming interdigit territory (upper middle and right panels in Fig. 4C). Following C59 treatment, *Grem1* expression is lost (lower left panel in Fig. 4C), but is re-expressed in the interdigit mesenchyme during recovery (lower middle and right panels in Fig. 4C). These results show that WNT signaling positively regulates the expression of the BMP ligand *Bmp2* and the antagonist *Grem1* in the distal and interdigit mesenchyme. It is important to consider that the transient reduction of LMP proliferation after C59 treatment (Fig. S7G) results in a smaller autopod primordia (lower right panels in Fig. 4A-C). This spatial constraint restricts the periodic digit-interdigit patterning, such that three or four digits form in most cases (86%) in C59-treated limb buds (at E10.5; Fig. 4D). This reduction in digit numbers agrees with previous studies showing that autopodial progenitor proliferation is linked to the number of digits formed (Alberch and Gale, 1983; Lopez-Rios et al., 2012).

**Fig. 5.**
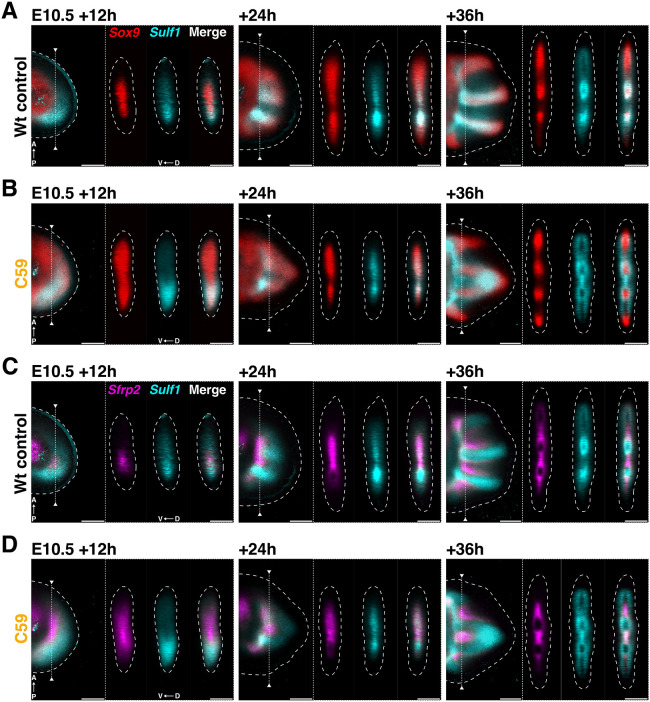
**The periodic expression of *Sulf1* and *Sfrp2* depends WNT signaling.** (A,B) Co-detection of *Sox9* (red) and *Sulf1* (cyan) to show the spatio-temporal expression dynamics in wild-type (A) and C59-treated (B) forelimb buds. Left panels: representative virtual mid-sections of the 3D images are shown. Right panels: virtual cross-sections of the dorso-ventral axis at the level indicated by a line in the left panels. (C,D) Co-detection of *Sfrp2* (red) with *Sulf1* (cyan) in the limb buds shown in A and B. *n*=3 biological replicates for all timepoints. Scale bars: 200 µm.

To date, the source and identity of WNT ligand(s) functioning in periodic digit-interdigit patterning has remained elusive. Notably, C59-mediated inhibition of WNT signaling identifies the *Wnt2b* ligand as a downregulated gene (Fig. 1E). This is likely of relevance to the establishment of the periodic digit pattern, as both *Wnt2b* and its paralogue *Wnt2* are expressed by the interdigit mesenchyme in wild types (Fig. S9B, Witte et al., 2009), which points to the interdigit as potential source of WNT ligands functioning in digit-interdigit patterning (Fig. 4E). This is consistent with the WNT activity being restricted to the interdigit mesenchyme during digit patterning either by (1) SOX9 suppressing *Wnt* ligand expression and/or (2) inhibition of WNT activity in the *Sox9*-expressing digit territory by negative modulation/antagonism of signal transduction (Raspopovic et al., 2014). Therefore, *Sox9-*positive cells could be a source of negative regulators acting in phase with *Sox9* after WNT signaling disruption. Indeed, gene expression profiling identifies that *Sulf1*, an extracellular modulator of WNT (and BMP) signaling, and the extracellular WNT antagonist *Sfrp2* are transiently upregulated together with *Sox9* in C59-treated forelimb buds (chondrogenesis in Fig. 1E, Fellgett et al., 2015; Kawano and Kypta, 2003; Otsuki et al., 2010; Sahota and Dhoot, 2009; Satoh et al., 2006). Multiple β-catenin peaks in the *Sulf1* and *Sfrp2* genomic landscapes point to negative transcriptional regulation by WNT/β-catenin (Fig. S8C,D). RNA-FISH shows that *Sox9*, *Sulf1* and *Sfrp2* are expressed in distinct spatial domains during wild-type autopod development (Fig. 5A,C). During digit-interdigit patterning, *Sulf1* expression (cyan) is restricted to the emerging *Sox9-*positive digit primordia (red; E10.5+24 h in Fig. 5A) and extends distally during digit ray elongation (E10.5+36 h in Fig. 5A). Conversely, *Sfrp2* expression (magenta) is restricted to the interdigit (+24 h in Fig. 5C) and its proximal expression boundary located at the presumptive wrist territory (+36 h in Fig. 5C). Inhibition of WNT signaling causes distal-anterior and dorso-ventral expansion of *Sox9* and *Sulf1* expression by ∼E11.0 (E10.5+12 h in Fig. 5B). Concurrently, *Sfrp2* expression expands precociously anterior within the distal limb bud mesenchyme (E10.5+12 h in Fig. 5D, compare with +24 h in Fig. 5C). During restoration of WNT signaling, the digit-interdigit periodicity of the *Sox9*, *Sulf1* and *Sfrp2* domains are re-established (+36 h in Fig. 5B,D). This shows that transiently blocking WNT signaling disrupts the periodic digit-interdigit patterning process, as *Sox9* expands at the expense of interdigit gene expression. However, digit-interdigit patterning is restored as WNT signaling recovers, which points to plasticity and/or robustness of the underlying digit-interdigit patterning system even if the digit-forming territory is smaller due to transiently reduced proliferation (Fig. S7G).

### Transient inhibition of WNT signaling during the establishment of the periodic digit-interdigit pattern

**Fig. 6.**
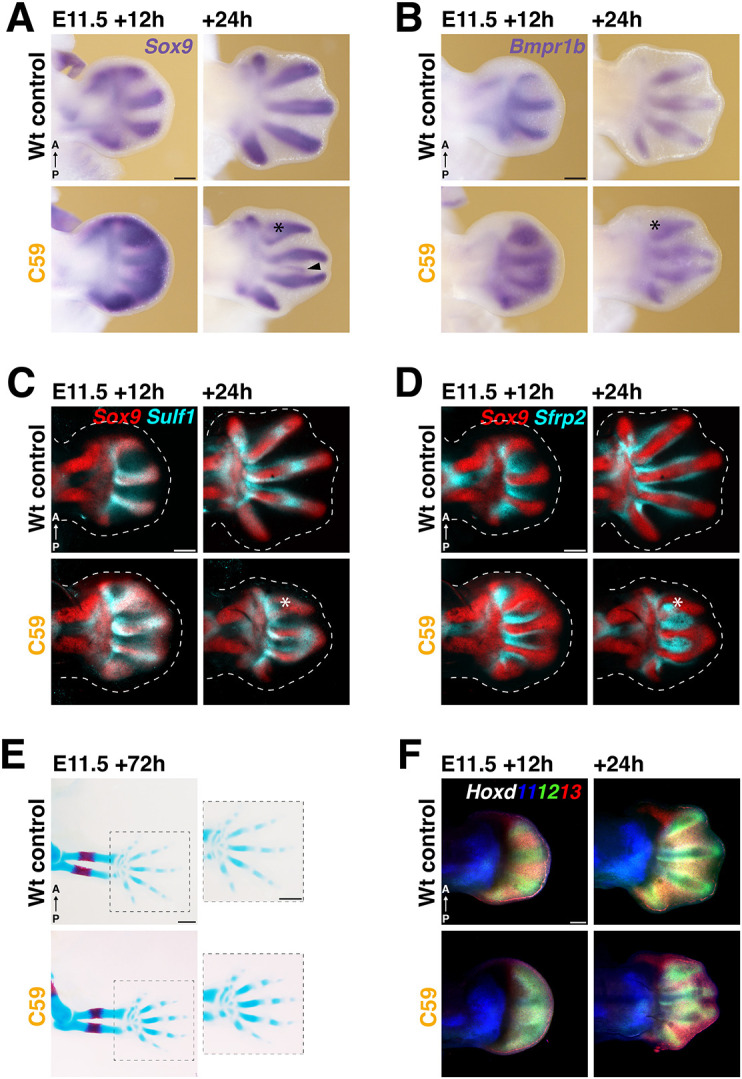
**Alteration of the digit and interdigit gene expression patterns following WNT inhibition during early digit ray development.** Wild-type control and C59-treated forelimb buds were analyzed at E11.5+12 and +24 h. (A,B) *Sox9* and *Bmpr1b* expression dynamics. (C) Co-detection of *Sox9* (red) and *Sulf1* (cyan) expression. (D) Co-detection of *Sox9* (red) and *Sfrp2* (cyan) in the same limb buds shown in C. Selected *z*-stack ranges are shown. Scale bars: 250 µm in A,B; 200 µm in C,D. (E) Limb skeletons of wild-type control and C59-treated embryos at ∼E11.5. Bone is visualized by Alizarin Red and cartilage by Alcian Blue. *n*=19 biological replicates were analyzed. Scale bars: 500 µm. (F) Co-detection of *Hoxd11* (blue), *Hoxd12* (green) and *Hoxd13* expression. The entire *z*-stack ranges are shown as maximum projections. Scale bar: 200 µm. *n*=3 biological replicates were analyzed for all genes and timepoints in A-D,F.

To gain insight into the spatiotemporal dynamics of digit-interdigit patterning during progressive digit ray formation (metacarpals), WNT signaling was disrupted at ∼E11.5. The observed downregulation and recovery of *Axin2* expression (Fig. S10A) establishes that the kinetics of transiently disrupting WNT signaling at this later stage is comparable to earlier limb bud stages (∼E10.5, Fig. 1B). C59 was injected at ∼E11.5, as inhibiting WNT signaling at this stage overlaps the period of AP digit patterning (Figs 1A and 6A), but precedes the onset of digit phalange patterning that is controlled by the PFR signaling center (≥E12.0 in mouse limb buds, Fig. 1A, Fig. S1B, Huang and Mackem, 2022). C59 injection at ∼E11.5 causes upregulation and expansion of *Sox9*, *Bmpr1b* and *Sulf1* into the interdigit territories and distal mesenchyme in comparison to wild-type controls (∼E12.0, left panels Fig. 6A-C). As WNT signaling is restored (Fig. S10A), the spatial expression of *Sox9*, *Bmpr1b* and *Sulf1* is again restricted to the digit ray primordia at ∼E12.5 (E11.5+24 h in Fig. 6A-C). However, aberrant digit curvatures and proximal bifurcations or broadening of the metacarpal base of the anterior digit 2 are also observed (asterisks in Fig. 6A-D, *n*=8/9). In addition, thin stripes of *Sox9* expression are detected in the interdigit territory (arrowhead in Fig. 6A, *n*=3/6). Effects on interdigit gene expression were assessed by analyzing the spatial distribution of *Sfrp*2 and *Bmp2* in wild-type control and C59-treated forelimb buds (Fig. 6D, Fig. S10B). In C59-treated forelimb buds, *Sfrp2* expression expands precociously distally within the interdigit, but is excluded from the distally expanded *Sox9* domain at ∼E12.0 (E11.5+12 h in Fig. 6D). In comparison to wild-type limb buds, *Bmp2* expression is lost from the distal mesenchyme but maintained in the proximal interdigit territory (∼E12.0, left panels in Fig. S10B). Both *Bmp2* and *Sfrp2* expression recover as WNT signaling is restored at ∼E12.5 (E11.5+24 h in Fig. 6D, Fig. S10B). Interestingly, the altered interdigit gene expression patterns are complementary to the metacarpal alterations (Fig. 6D), which appear transient as the definitive metacarpal morphology and pentadactyly are preserved (*n*=18/19, lower panels in Fig. 6E).

Distal Hox genes have been proposed to modulate the digit wavelength as part of the Turing digit-interdigit patterning system (Raspopovic et al., 2014; Sheth et al., 2012). RNA-FISH analysis of *Hoxd11*, *Hoxd12* and *Hoxd13* in wild-type forelimb buds reveals that *Hoxd12* expression is highest in digit primordia, while *Hoxd13* expression is highest in interdigits at E12.0-E12.5 (top panels in Fig. 6F, Fig. S10C). After C59 treatment at ∼E11.5, *Hoxd12* expression expands into the interdigit mesenchyme, while *Hoxd13* expression is reduced (E11.5+12 h, C59 panel in Fig. 6F; left panels Fig. S10D). After WNT restoration, the digit-interdigit expression of *Hoxd12* and *Hoxd13* recover and is comparable to wild-type limb buds (E11.5+24 h in Fig. 6F, Fig. S10C,D). Together, this analysis shows that C59-mediated transient inhibition of WNT signaling during early limb bud outgrowth and later during digit-interdigit patterning significantly impacts the expression of key WNT targets genes as part of the early limb bud signaling system (Bénazet et al., 2009) and periodic digit-interdigit patterning (Raspopovic et al., 2014; Sheth et al., 2012). As WNT signaling is restored, the gene expression and digit-interdigit patterns largely recover, revealing significant robustness and/or plasticity of the limb bud and digit-interdigit patterning systems.

## DISCUSSION

In this study, we use IP injection of the small molecule C59 into pregnant female mice to rapidly and transiently disrupt WNT signaling during limb bud development. This analysis establishes that (1) WNT/β-catenin signaling is required to maintain and propagate the self-regulatory SHH/GREM1/AER-FGF signaling system; (2) proliferative expansion of LMPs occurs during limb bud outgrowth and autopod formation; (3) there is restriction of *Sox9*-expressing progenitors to the chondrogenic core mesenchyme and developing digit primordia; and (4) initial establishment of the periodic digit-interdigit pattern occurs during early autopod development.

Overall, the rapid and transient alterations observed in this study point to efficient removal and restoration of extracellular WNT ligands, which is also the case for extracellular GREM1. Both WNTs and GREM1 bind to heparan sulfate proteoglycans, which can promote both ligand stabilization and internalization (Capurro et al., 2008; Christianson and Belting, 2014; Mii and Takada, 2020; Tatsinkam et al., 2015). Thus, the swift drop in extracellular WNT ligands (and GREM1) in the absence of newly secreted proteins (+6, +12 h) is likely a consequence of endocytosis, potentially mediated by HSPGs (Capurro et al., 2008). The disruption of WNT signaling is paralleled by equally fast alterations in target gene expression. This is a likely the consequence of depleting β-catenin from functionally relevant CRMs following inhibition, which points to direct regulation of target genes by WNT signal transduction. Regulation of gene expression by canonical WNT/β-catenin signal transduction depends interactions with co-factors, such as TCF/LEF, and assembly of a large multiprotein complexes at CRMs (Cadigan and Waterman, 2012; Söderholm and Cantù, 2021). Interaction of β-catenin with these multiprotein complexes at WNT-responsive elements promotes transcription (Söderholm and Cantù, 2021). It is likely that the C59-mediated WNT signaling disruption and decline of nuclear β-catenin affect the enhancer-promoter interactions, while multiprotein complexes, including TCF/LEF and other co-factors, remain bound to *cis*-regulatory elements. In such a poised state, restoration of WNT signaling would enable re-establishment of enhancer-promoter interactions and periodic gene expression, as is seen during recovery (this study). Notably, there is significant plasticity in spatial gene regulation, as inhibition of WNT signaling causes rapid and complementary expression changes in genes expressed in digit (expanded) and interdigit (reduced) territories. This is in agreement with a key regulatory role for the WNT pathway in digit-interdigit patterning (Raspopovic et al., 2014). In addition, transient inhibition of WNT signaling (≤E10.75 to ≤E11.5) reduces limb bud cell proliferation, which results in formation of smaller autopod primordia. The delayed onset of the periodic digit-interdigit pattern in C59-treated limb buds occurs with a periodicity similar to wild type. In agreement the expression of distal Hox genes, which regulate the wavelength of digit periodicity, is restored in parallel to WNT signaling (Sheth et al., 2012). Therefore the resulting oligodactylies with three or four digits, or fused middle digits are not due to developmental heterochrony, but rather due to the periodic digit-interdigit patterning acting on the spatially constraint digit territory, which might be relevant to congenital and evolutionary digit reductions (Cooper, 2015; Lopez-Rios et al., 2014; Sheth et al., 2012).

**Fig. 7.**
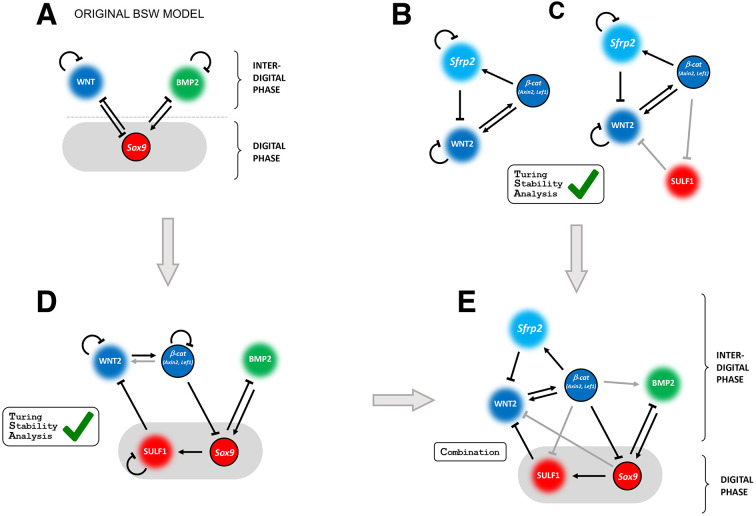
**Evaluation of the WNT signaling pathway components in an expanded BMP-SOX9-WNT Turing network.** (A) The BMP-SOX9-WNT (BSW) Turing model according to Raspopovic et al. (2014). (B) Modelling of a three-node circuit involving WNT2, β-catenin and SFRP2 demonstrates that this circuit can act as a classical activator-inhibitor module with all three components in-phase (in the interdigital tissue). (C) Extension of the three-node network (B) to incorporate the WNT modulator SULF1 expressed in the digit mesenchyme (out-of-phase with SFRP2) as a negative modulator of WNT2. This extended network also functions as a Turing network. (D) Extension of the original BSW model (A), to include SULF1 downstream of *Sox9* to repress diffusible WNT2 ligand in the digit mesenchyme (in gray). This model also includes β-catenin acting in a cell-autonomous manner. Mathematical simulations using RDNets show that this extended BSW network functions as a substrate-depletion Turing system (rather than an activator-inhibitor model). BMP2 is the substrate in this system. (E) Numerical simulations of the four-node Turing circuit (C) in combination with the five-node circuit (D) establish that this extended network (six nodes in total) can also function as a Turing circuit. Black arrows indicate that the interaction has been tested by computational modelling; gray arrows indicate hypothetical interactions compatible with the model.

The recovery from early inhibition of WNT signaling also covers the period during which the self-regulatory limb bud signaling system transitions to a periodic digit-interdigit patterning system. This transition is paralleled by genes required for autopod development becoming independent of SHH (Panman et al., 2006) and by progressive shut-down of the SHH/GREM1/AER-FGF signaling system (Farin et al., 2013; Verheyden and Sun, 2008). During this transition, interactions between WNT, BMP signaling and the *Sox9* transcription factor have been proposed to initiate the periodic digit-interdigit pattern system with characteristics of a self-organizing Turing mechanism (Fig. 7A, Raspopovic et al., 2014). Both genetic inactivation of β-catenin during autopod development and C59-mediated WNT inhibition cause expansion of the *Sox9* expression domain and prevent timely establishment of the periodic digit-interdigit pattern (this study). Although C59 blocks both canonical and non-canonical WNT signaling (Proffitt et al., 2013), genetic analysis reveals the requirement of β-catenin-mediated canonical WNT signaling during early limb bud development and periodic digit-interdigit patterning (this study). This is corroborated by inactivation of the non-canonical *Wnt5a* ligand, which does not alter the establishment of the digit-interdigit pattern (Yamaguchi et al., 1999). These and other studies indicate that canonical WNT/β-catenin signal transduction is required for initiation of the periodic digit-interdigit pattern, while non-canonical WNT signaling functions subsequently in regulating growth and elongation of digit primordia as part of a convergent-extension mechanism (Parada et al., 2022).

It has been shown that a fundamental property of Turing systems is their ability to self-reorganize after disruption, often resulting in curved stripes and loops. This is associated with the intrinsic self-organizing behavior of Turing systems, whereby the positions and spacing of the periodic pattern arise spontaneously and are maintained in space. Experimental perturbation in different developmental systems have shown how Turing patterns self-reorganize the pigmentation stripes in fish (Kondo and Asai, 1995), rodent fur stripe patterns (Johnson et al., 2023), rugae formation during palate patterning (Economou et al., 2012) and avian tracheal cartilage ring patterns (Kingsley et al., 2023 preprint). In this study, transient inhibition of WNT signaling allowed evaluation of the self-reorganization potential of periodic digit-interdigit pattering in mouse limb buds. Transiently blocking WNT signaling at E10.5 prevents the initial establishment of the *Sox9* periodicity; however, the periodic pattern emerges upon restoration of WNT signaling, which results in variable curved digits as expected from disrupting a Turing system. Transient inhibition of WNT signaling at ∼E11.5 causes expansion of *Sox9* into the interdigit territory, which transiently perturbs the pattern. This causes transient bifurcations of digit gene expression domains, ectopic *Sox9* stripes and curved morphologies, together with altering digit and interdigit gene expression patterns. These alterations and the subsequent recovery of periodicity point to substantial plasticity of digit-interdigit patterning system, in line with an underlying Turing system (Raspopovic et al., 2014; Sheth et al., 2012). After establishment of the initial digit-interdigit pattern, cartilage condensation and distal elongation of digit primordia are controlled by *Wnt5*-dependent convergent-extension movements that couple PFR formation with digit elongation and phalange patterning (Parada et al., 2022). Finally, recent analysis of phalange patterning in chicken and mouse limb buds shows that establishment of the periodic joint and phalanx pattern is controlled by a Turing mechanism involving the BMP ligand GDF5 and the BMP antagonist Noggin (Grall et al., 2024).

Several WNT ligands, WNT antagonists and signaling modulators are expressed by the limb bud mesenchyme and ectoderm during establishment of the digit-interdigit pattern (Geetha-Loganathan et al., 2008; Murphy et al., 2022; Witte et al., 2009). It has been suggested that WNT signaling is required for symmetry breaking (Raspopovic et al., 2014; Fig. 7A, Fig. S11A). In agreement, this study shows that the *Wnt2*/*Wnt2b* ligands and the WNT antagonist *Sfrp2* are expressed in forming interdigit territories (Figs 4E and 5C), while the signaling modulator *Sulf1* is expressed by *Sox9*-positive LMPs (Fig. 5A). These expression patterns permitted modelling of a variety of network topologies using the RDNets tool (Marcon et al., 2016) and stability analysis showed that a simple circuit incorporating WNT2, β-catenin and SFRP2 can function as a classic activator-inhibitor Turing-type circuit (Fig. 7B). The relationship between these molecules and a classic activator-inhibitor circuit is illustrated in Fig. S11B. It is possible that this WNT-based circuit is initiated during symmetry breaking and functions in establishing the interdigit pattern. Furthermore, negative modulation of WNT signaling by SULF1 downstream of SOX9 could also contribute to restrict WNT activity to the interdigit territory (Fig. 7C,D; Fig. S11C). The BSW Turing model predicts that BMP signaling from the interdigit mesenchyme promotes *Sox9* expression in the mesenchyme destined to form digit territories. Finally, combining these two additional Turing circuits generates a Turing pattern in which the signal modulator SULF1 and antagonist SFRP2 are expressed out-of-phase in digit and interdigit territories, respectively, to modulate and restrict WNT signaling (Fig. 7E, Fig. S11D). Genetic analysis of *Sfrp1* and *Sfrp2* in mouse embryos revealed functional redundancy, but inactivation of both Sfrp genes causes a pleiotropic phenotype that includes stunting and broadening of limb skeletal elements and preaxial polydactyly (Satoh et al., 2006). Similarly, *Sulf1* and *Sulf2* regulate several developmental signaling pathways by cleaving sulfate groups from heparan sulfate proteoglycans (Ai et al., 2003; Fellgett et al., 2015; Otsuki et al., 2010; Sahota and Dhoot, 2009). Loss-of-function analysis reveals their requirement for modulating endochondral bone differentiation (Ratzka et al., 2008), which is not informative with respect to digit-interdigit pattern. In summary, the present study based on transient inhibition of WNT signaling reveals the substantial plasticity of the periodic digit-interdigit patterning system. This study, together with the consecutive Turing mechanism that controls phalange-joint segmentation, indicates that spatial modulation of signals by antagonists contributes both robustness and evolutionary plasticity (Grall et al., 2024).

## MATERIALS AND METHODS

### Animals

#### Ethics statement and approval of all animal experimentation

All studies involving mice were performed in accordance with national laws and approved by the national and local regulatory authorities, as mandated by law in Switzerland. All proposed animal studies were approved by the Regional Commission on Animal Experimentation and the Cantonal Veterinary Office of Basel (national license 1951) in accordance with Swiss laws and the 3R principles.

#### Mouse strains and embryos

In line with the refine and reduce 3R principles, all strains were bred into a Swiss Albino (*Mus musculus*) background, as only robust phenotypes manifest in this strain background and the numbers of embryos and litter sizes are large (≥12-15 embryos per pregnant females). Embryos of both sexes at the developmental ages indicated were used for experimental analysis. For conditional β-catenin inactivation, TMCRE*Ctnnb1*^Δc/+^ mice (tamoxifen-inducible CRE; Hayashi and McMahon, 2002) were crossed to *Ctnnb1*^Δc/Δc^ mice (Catnnb1lox mice – B6.129-Ctnnb1^tm2Kem^/KnwJ; Huelsken et al., 2001). For tamoxifen injections, the tamoxifen stock solution (Sigma-Aldrich T5648) was prepared by dissolving tamoxifen powder in corn oil (Merck C8267) at a concentration of 20 mg/ml. Per mouse (35-40gr), one dose of 5 mg tamoxifen in 250 µl corn oil solution was injected intraperitoneally to induce conditional gene inactivation.

### Small molecule inhibition of WNT signaling

Wnt-C59 (Tocris, 5148-10 mg), a potent porcupine inhibitor that blocks secretion of WNT ligands (Proffitt et al., 2013; Szenker-Ravi et al., 2018), was prepared as a 5 mg/ml stock solution in DMSO (Sigma-Aldrich, D8418-250 ml). Before injection, the C59-stock solution was diluted in vehicle solution [1×PBS, 0.5% methylcellulose (Sigma-Aldrich, M0262-100G), 0.01% Tween-20 (Sigma-Aldrich, 93773-1 kg) and 5% DMSO; Szenker-Ravi et al., 2018] to the required working concentration. For DMSO (wild-type controls) and C59 injections, pregnant Swiss Albino females were weighed in the morning of gestational days 10 or 11 prior to timed IP injection. The C59 and DMSO injection volumes were calculated in relation to bodyweight as follows. For wild-type controls, 10% DMSO (in 1×PBS) was injected intraperitoneally in a volume of 10 µl per gram of bodyweight. To inhibit WNT signaling; 10 µg/10 µl C59 per gram bodyweight was injected intraperitoneally. After IP injection, the embryos are isolated at the indicated timepoints.

### Embryo collection and staging

For whole-mount *in situ* hybridization, RNA-FISH or immunostaining embryos were collected in ice-cold PBS and subsequently fixed in 4% PFA overnight. After washing in PBS, embryos were dehydrated stepwise into 100% methanol and stored for at least 1 day at −20°C. Wild-type limb buds were staged by somite counting and limb bud shape. Due to the requirement of WNT signaling for somitogenesis, C59-treated (and following conditional β-catenin inactivation) embryo mutant limb buds were staged (1) according to the time elapsed at the time of isolation since the C59 or tamoxifen IP injection; and (2) by comparing limb bud sizes and shapes to wild-type limb buds. Biological replicates were generated by analyzing forelimb buds from different DMSO-injected wild-type and C59-treated embryos. For each condition and time point, the biological replicates included embryos from at least three different litters at all timepoints. To avoid potential left-right bias, the limb buds analyzed were chosen randomly.

### Limb skeletal preparations

Embryos were collected in the late afternoon of embryonic day 14, washed in 1×PBS and fixed in 95% ethanol at room temperature overnight. After fixation, embryos were stained for 24 h in 0.03% (w/v) Alcian Blue (Sigma-Aldrich, A3157) diluted in 80% ethanol and 20% glacial acetic acid (Sigma-Aldrich, 100063). They were then washed for 24 h in 95% ethanol. Subsequently, embryos were pre-cleared for 30 min in 1% KOH (Sigma-Aldrich, 105033) and counterstained with 0.005% (w/v) Alizarin (Sigma-Aldrich, A5533) in 1% (w/v) KOH. Embryos were then cleared in glycerol/1% KOH by stepwise increases in the glycerol concentration (20%, 40%, 60% and 80% glycerol) and preserved in 80% glycerol in water. Alcian Blue detects cartilage, while Alizarin Red stains ossified bone. Analysis was conducted on at least three embryos per genotype (see Figure legends).

### Whole-mount RNA *in situ* hybridization using BM-Purple staining

A standard protocol for whole-mount RNA *in situ* hybridization was used (Haramis et al., 1995). Following rehydration, embryos underwent bleaching in 6% hydrogen peroxide that was followed by digestion with 10 μg/ml proteinase K (duration adjusted based on embryonic stage and adapted for genes expressed by the limb bud ectoderm). After prehybridization at 65°C for at least 3 h, embryos were incubated overnight at 70°C in hybridization solution containing 0.2-1 μg/ml heat-denatured antisense riboprobe to detect the transcripts of interest. The next day, embryos were extensively washed and non-hybridized riboprobe digested with 20 μg/ml RNase at 37°C for 45 min. After additional washes and pre-blocking, embryos were incubated overnight at 4°C with anti-digoxigenin antibody (1:5000, Roche, 11093274910). After several washes to remove excess antibodies, RNA-riboprobe hybrids were visualized by incubation in BM Purple staining solution (Roche, 11442074001). Development of the BM Purple signal stopped during exponential phase before reaching saturation.

### Fluorescent RNA *in situ* hybridization (RNA-FISH)

An improved whole-mount RNA-FISH protocol that is optimized for reducing tissue autofluorescence has been described previously (Morabito et al., 2023). This protocol was used in combination with mouse HCR probes (Molecular Instruments), enabling analysis of multiple gene expression patterns in a single biological replicate by using specific fluorophores to detect individual probes. Briefly, rehydrated and stage-matched limb buds were subjected to photochemical bleaching (2×30 min at room temperature) followed by incubation in detergent solution for 2 h at 37°C. To detect gene expression, limb buds were hybridized with specific mouse HCR probes (Molecular Instruments) for 16 h. To prevent low level expression (e.g. *Wnt2*), the HCR probe concentration was doubled and the incubation time extended to 20-24 h. Signal amplification was carried out as described in the step-by-step protocol, but for difficult to detect transcripts, the amplifier concentration was doubled and the incubation time increased to 24 h. During washing in 5×SSCT, nuclei were counterstained with DAPI (1:1000, Sigma-Aldrich, D9542). In order to wash away the high salt concentration, limb buds were washed in 1×PBS for 2 min and then immersed in the fructose-glycerol clearing solution. Samples were cleared for at least 24 h prior to imaging.

### Whole-mount immunofluorescence

Rehydrated age-matched limb buds were briefly washed in PBS, then permeabilized and unspecific binding blocked in 5×SSCT, 1% Triton and 10× donkey serum for ≥3 h with gentle shaking at room temperature. The following primary antibodies were used: goat-anti-GREM1 (1:50, R&D Systems, AF956) and rabbit-anti-pSMAD1.5.9 (1:200, Cell Signaling Technology, 13820S). Apoptotic cells were detected using rabbit anti-cleaved caspase 3 antibodies (Asp175, 1:200, Cell Signaling Technology, 9661S). Limb buds were incubated with the respective antibodies diluted in 5×SSCT, 1% Triton and 10× donkey serum for 72 h at room temperature with gentle shaking. The limb buds were washed in 5×SSCT, 1% Triton and 10× donkey serum for 4 h with several changes. Depending on the multiplexed primary antibodies, individual or a combination of the following secondary antibodies were used: donkey anti-rabbit-488 (1:250, Jackson, 711-545-152), donkey anti-rabbit-555 (1:250, Invitrogen, A31572), donkey anti-rabbit 647 (1:250, Invitrogen, A31573) and anti-goat 647 (1:250, Invitrogen, A21447). Limb buds were incubated with secondary antibodies diluted in 5×SSCT, 1% Triton and 10× donkey serum containing DAPI (1:1000) at room temperature with gentle shaking. After washing in 5×SSCT, 1% Triton and 10× donkey serum, limb buds were quickly washed in 1×PBS and the refractory index adjusted as described for above for RNA-FISH.

### Image acquisition and data processing

Images were acquired using a confocal spinning disc microscope with a 10× objective (10×/0.45 CFI Plan Apo), a confocal spinning disc scan unit (Yokogawa Spinning Disk CSU-W1-T2) and a Nikon Ti-E, Hamamtsu Flash 4.0 V2 CMOS camera. Images intended for 2D maximum intensity projections were acquired with a 5 µm *z*-step size, while for 3D reconstruction purposes the step size was reduced to 1 µm. For 2D maximum projections raw images were processed using FIJI. The entire or a specific stack-range was selected and stacked (“Z Project...”, “start=X stop=Y projection=[Max Intensity]”). As autofluorescence was much reduced by the photochemical bleaching prior to fluorescent detection of gene expression patterns, only brightness and contrast adjustments were made for RNA-FISH analyses. Three-dimensional images constructed from 2D image stacks were generated using the IMARIS software. Raw files were therefore converted to the .ims format, and brightness and contrast values adjusted. Virtual sections were obtained from 3D images using either the “Section” or “Clipping Plane” tools.

### RNA-sequencing time course

Two to three pairs of stage-matched forelimb buds from DMSO or C59-treated embryos were fine dissected and washed in fresh ice-cold PBS, immediately transferred to RNA-later (Sigma-Aldrich R0901) and stored overnight at −4°C. The next day, samples were transferred to −80°C for long-term storage. For each biological replicate, total RNA was isolated from one pair of forelimb buds using the RNeasy Kit (Qiagen, 74104) following the manufacturer's instructions. After performing QC analysis on a Fragment Analyzer (Advanced Analytical Technologies), RNA was stored at −80 until shipping to the company Novogene (UK). Standard Illumina library preparations and sequencing was performed by Novogene. After quality control, libraries were sequenced as PE150 on a NovaSeq6000.

### ChIP-seq analysis

Dissected forelimb bud tissues were crosslinked using 1% formaldehyde/1×PBS at room temperature for 12 min and crosslinking was stopped in 125 mM glycine solution. Per biological replicate, 20 forelimb buds were pooled for analysis. For β-catenin ChIP, an initial crosslinking step was performed using DSG (disuccinimidyl glutarate, Sigma-Aldrich, 80424) for 40 min at room temperature as described previously (Sheth et al., 2016). After lysis in hypertonic buffer, chromatin fragments were generated by sonication. Immunoprecipitation was performed at 4°C overnight using polyclonal anti-β-catenin antibodies (Invitrogen, 71-2700; 5 μg per sample) or anti-histone H3(acetyl K27) antibodies (ChIP Grade; Abcam, ab4729; 5 μg per sample). The chromatin complexes were immunoprecipitated using magnetic beads (Fisher Scientific, 11202D). After washing beads in RIPA buffer, DNA was eluted from beads followed by overnight reverse cross-linking. Libraries were generated using the next-generation library preparation kit from Takara Bio (Japan) according to manufacturer instructions and the ChIP-Seq libraries were sequenced using a NextSeq instrument (Illumina).

### Comparative cell cycle analysis of DMSO and C59-treated forelimb buds

Forelimb buds were dissected at four timepoints after IP injection from DMSO- (wildtype control) and C59-treated embryos. Forelimb buds from one or two litters (*n*=18-30 pairs) constituted one biological replicate. Single-cell suspensions were prepared by incubating limb buds with 2 mg/ml Collagenase D (Roche, 11088882001) for 20 min at 37°C, with gentle pipetting every 5 min. The reaction was stopped with ice-cold 10% FBS in PBS. Cells were filtered (Merck, BAH136800040), counted and washed in PBS. Fixation was performed by dropwise addition of −20°C ice-cold 70% ethanol while vortexing. After 30 min on ice, cells were washed twice in PBS and stained with 0.75 µg/ml DAPI (per two million cells). After two more PBS washes, cells were resuspended and filtered through a cell strainer (Falcon 352235). DAPI content per cell was measured on a CytoFLEX S (Beckman Coulter) and data analyzed using FlowJo (BD Life Sciences) with the built-in Watson (Pragmatic) algorithm to determine cell cycle phases. Plots and statistical analyses were carried out using the Prism software (v10.2.3). For each timepoint, *n*=4 wild-type controls and *n*=5 C59-treated biological replicates were analyzed, which revealed statistically highly significant differences using the unpaired non-parametric Mann–Whitney test.

### Bioinformatics analysis

The calculations were performed using the facilities of the Scientific IT and Application Support Center of the EPFL.

### RNA-seq analysis

Low-quality bases and Truseq adapters were removed with Cutadapt v1.16 (Martin, 2011) using the following sequences: -a GATCGGAAGAGCACACGTCTGAACTCCAGTCAC and -A GATCGGAAGAGCGTCGTGTAGGGAAAGAGTGTAGATCTCGGTGGTCGCCGTATCATT -q 30-m 15. Reads were mapped on mouse genome assembly mm10 with STAR (Dobin et al., 2012) version 2.7.0e using ENCODE parameters and custom gtf (https://doi.org/10.5281/zenodo.7510406). Counts and coverage were computed at the same time. The values for fragments per kilobase of transcript per million mapped reads (FPKM) were computed using the cufflinks version 2.2.1 program (Trapnell et al., 2010) with the following parameters: --max-bundle-length 10000000--multi-read-correct --library-type “fr-unstranded” -b /home/ldelisle/genomes/fasta/mm10.fa --no-effective-length-correction -M MTmouse.gtf -G custom.gtf.

Average coverage between replicates was computed using the bigwigAverage from deepTools version 3.5.5. FPKM values excluding genes on chromosomes X, Y and M were transformed with log2 (1+FPKM). PCA was computed (centered, non-scale) using a selection of 2000 genes with the highest variance. The same matrix was used to compute Spearman's correlation between samples and clustered with the ward.D2 method. Counts from STAR were restricted for protein-coding genes, and genes on chromosomes X, Y and M were excluded. Differential analysis was performed with DESeq2 version 1.34.0 (Love et al., 2014) on R 4.1.3 for each experimental timepoint after comparing three each of wild-type and C59-treated replicates. DEGs with an adjusted *P*-value below 0.05 and an absolute log2 fold-change above 1 were selected. All genes significantly altered minimally at one timepoint between wild-type and C59- treated limb buds were considered for clustering. The log2 fold-change values estimated by DESeq2 were centered, scaled (per gene) and used to compute the Pearson's correlation between genes. Genes were clustered using the ward.D2 method and the tree was cut into three clusters. The log2 fold-changes displayed in Fig. 2 are DESeq2 log2 fold-change (before scale transformation). GO analysis was performed using GOseq version 1.56.0 (Young et al., 2010) including the genes of each cluster and using all protein-coding genes as background.

### ChIP-seq analysis

Low quality bases and Truseq adapters were removed with Cutadapt v1.4 (Martin, 2011) using the following sequences: -a GATCGGAAGAGCACACGTCTGAACTCCAGTCAC and -A GATCGGAAGAGCGTCGTGTAGGGAAAGAGTGT -q 30 -m 15. Filtered reads were mapped with Bowtie2 version 2.4.5 (Langmead and Salzberg, 2012) with default parameters. Alignments with MAPQ below 30 were filtered out with samtools version 1.16.1 (Danecek et al., 2021). Peaks were called and coverage was performed with macs2 version 2.2.7.1 (Zhang et al., 2008) using the following parameters: -g 1870000000 --call-summits --format BAMPE -B, input was not used.

Coverage was normalized by the million fragments after filtering. Average coverage between replicates was computed with bigwigAverage from deepTools version 3.5.5 (Ramírez et al., 2016). Consensus peaks were obtained as follows.

(1) Peaks were called using all replicates with equal contributions as follows. Duplicates were removed from the BAM files of each replicate with Picard version 2.27.4 (http://broadinstitute.github.io/picard/). Each BAM was subsampled to identify the number of reads of the smallest BAM. Peaks were called using all subsampled BAM with the same criteria as for individual replicates.

(2) Only peaks with overlapping summits in both replicates were kept. In particular, individual narrowPeaks were overlapped using BEDTools multiinter version 2.30.0 (Quinlan and Hall, 2010) and only intervals present in both replicates were kept. The narrowPeak file of step 1 was intersected with the output of multiinter and was filtered in order to keep only narrowPeaks with summits overlapping the output of multiinter. Consensus β-Catenin ChIP-seq summits in wild types were filtered to remove summits overlapping the ENCODE blacklist (Amemiya et al., 2019). Any summit located less than 1 kb from a transcriptional start site (TSS) of the custom GTF (see RNA-seq analysis) was considered a TSS. Others were considered as distal peaks.

(3) Heatmaps centered on consensus β-catenin summits at TSS or distal regions were generated using deeptools version 3.5.5 (Ramírez et al., 2016) with a bin size of 10 bp at ±3.5 kb. These summits were used to identify *de novo* transcription factor binding motifs in a 200 bp window using Homer version 4.11 (Heinz et al., 2010). VISTA enhancers coordinates were downloaded from https://enhancer.lbl.gov/ and lifted over from mm9 to mm10, and intersected with the consensus β-catenin summits.

### Gene network analysis

A protein-protein interaction network was generated from 73 genes downregulated at 6 h after C59 IP injection (Table S4) using the online tool “NetworkAnalyst” (Zhou et al., 2019). The following settings were used: String database, confidence score 850, no experimental evidence, First order-network. The Subnetwork1 included 24 interconnected seeds of which *Tac1*, *Ccl28*, *Igf1*, *Bdnf* and *Ngf* (not relevant for this study) were removed for representation purposes.

### Turing network simulations

To explore whether hypothetical regulatory networks were capable of creating Turing patterns, we used two approaches. For Fig. 7, we used the public online tool RDNets (Marcon et al., 2016), which allows the user to graphically specify network topologies, and performs mathematical analysis to determine if a network is compatible with the Turing conditions. Topologies can be fully specified (both the sign of regulatory links and whether each node is diffusible or not) or ambiguities can be allowed, and the program will explore different options. Due to its complexity, for the full combined network in Fig. 7E, we performed numerical simulations in a spatial domain using the modelling platform LimbNET (Matyjaszkiewicz and Sharpe, 2024 preprint) instead of an analytical solution. We manually found suitable parameter values that successfully created a Turing pattern.

## Supplementary Material



10.1242/develop.204606_sup1 Supplementary information

Table S1. Temporal profile of differentially expressed genes. DEGs identified at 1, 6, 12, 18 and 24hrs after C59 injection. Linked to Fig. 1, 2A.

Table S2. DEG fold-change expression profiles over time. DEGs were split into three clusters based on their temporal fold-change expression profiles. Linked to Fig. 1D.

Table S3. DEGs selected for line plots analysis of the relevant pathways. Linked to Fig. 1E.

Table S4. DEGs at the E10.5 +6hrs timepoint selected for network analysis. Linked to Fig. 2A.
