# Supplementary material for: WNT signaling coordinately controls mouse limb bud outgrowth and establishment of the digit-interdigit pattern
Source: Development. 2025 Jun 10;152(11):dev204606. doi: 10.1242/dev.204606 (PMC12188246; doi:10.1242/dev.204606)
Supplement: Supplementary information [file develop-152-204606-s1.pdf]

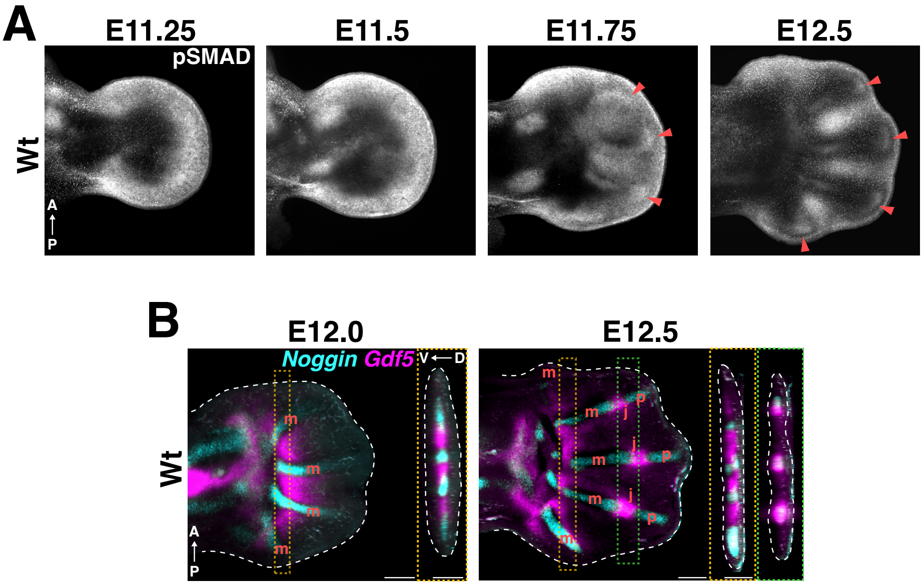

Fig. S1. The phalange forming regions become molecularly apparent ~E11.75 and the first joint and phalange have formed by E12.5 in wild-type forelimbs. (A) Developmental series of the pSMAD (=pSMAD1,5,9) distribution using wholemount immunofluorescence analysis of wild-type forelimb buds between E11.25 and E12.5. Red arrow heads point to the appearance of the PFR pattern. By E11.75, crescents of high pSMAD expression in the distal tip of forming digit rays reveal the presence of PFRs that become well separated at E12.5. (B) Whole mount RNA-Fish of *Noggin* (cyan) and *Gdf5* (magenta) in wild-type limb buds show that up to E12.0 metacarpals (m) are forming but the future joint marker *Gdf5* is expressed in the proximal part of the interdigit domains. By E12.5, *Gdf5* is also expressed in developing joints (j), which divide the *Noggin*+ digit rays into metacarpals (m) and the first phalanges (p). RNA-FISH and immunofluorescence images are shown as maximum intensity projection of a selected Z-stack range. n=3 independent developmental timeseries were analyzed for panels A and B. A=anterior, P=posterior, D=dorsal, V=ventral.

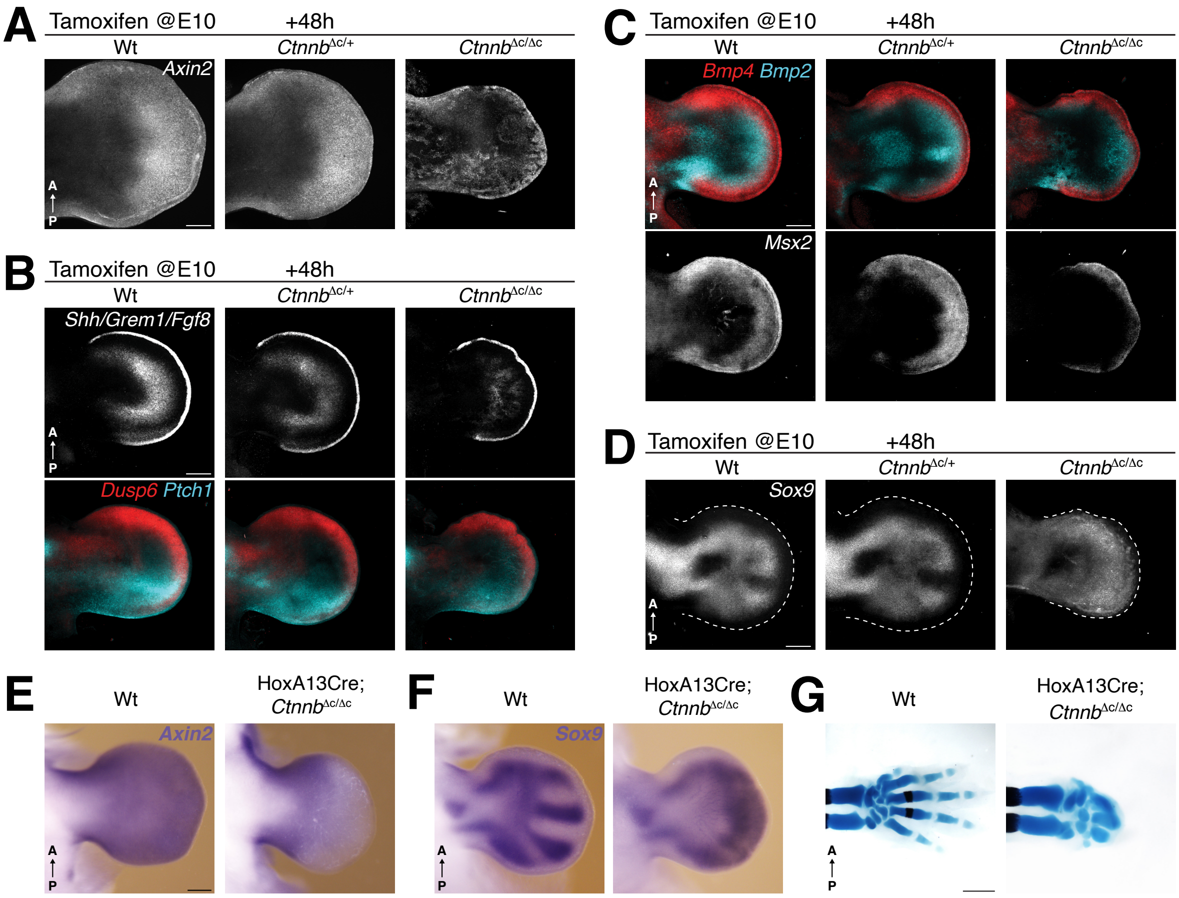

Fig. S2. Temporal kinetics of conditional genetic β-catenin gene inactivation in mouse forelimb buds. (A-D) The expression of key genes functioning in growth, patterning and chondrogenesis were assessed by RNA-FISH in wild-type (Wt), heterozygous (*Ctnnb1*$^{\Delta c/+}$) and homozygous (*Ctnnb1*$^{\Delta c/\Delta c}$) forelimb buds at 48hrs after tamoxifen injection at ~E10. (A) Expression of the WNT transcriptional sensor *Axin2* is significantly reduced but not lost at E10+48hrs. (B) Upper panels: *Shh*, *Grem1* and AER-*Fgf8* expression were detected in the same channel. While *Shh* expression is significantly reduced/lost, *Grem1* expression is patchy and AER-*Fgf8* expression is somewhat reduced. Lower panels: in the same forelimb buds, the transcriptional sensors *Ptch1* (cyan, for SHH) and *Dusp6* (red, for AER-FGF signaling) were assessed. (C) Upper panels: *Bmp2* (cyan)and *Bmp4* (red) expression in forelimb buds. Both *Bmp2* and *Bmp4* expression are reduced but not lost in *Ctnnb1*$^{\Delta c/\Delta c}$ forelimb buds. Lower panels: in the same forelimb buds, *Msx2* expression was assessed. *Msx2* expression is reduced and distalized in *Ctnnb1*$^{\Delta c/\Delta c}$ forelimb buds. (D) The *Sox9*

Development • Supplementary information

expression domain is significantly expanded in *Ctnnb1*$^{\Delta c/\Delta c}$ forelimb buds. n=3 independent biological replicates were analysed for all genotypes and stages. All images are shown as maximum intensity projection of the entire Z-stack range. Forelimb buds are oriented with anterior to the top and posterior to the bottom. Scale bars: 200µm. (E) *HoxA13*Cre-mediated *β-catenin* inactivation in the distal limb bud mesenchyme. In comparison to controls, *Axin2* is downregulated in the developing handplate (reminiscent of the *HoxA13* expression domain. (F) *Sox9* is expressed by the distinct digit ray progenitors in wild-type forelimb buds (left panel). In the *β-catenin* deficient hand plates, the characteristic *Sox9* expression in the periodic digit domains is absent as *Sox9* expression is expanded in the distal mesenchyme (right panels). (G) Comparison of the developing wild-type (left) and *HoxA13*Cre *β-catenin* deficient autopod skeleton (right). In mutant forelimbs only 2-3 malformed and distally fused digits have formed at E14.5. n≥3 independent biological replicates were analysed for all probes and developmental stages. Limb buds are oriented with anterior to the top and posterior to the bottom.

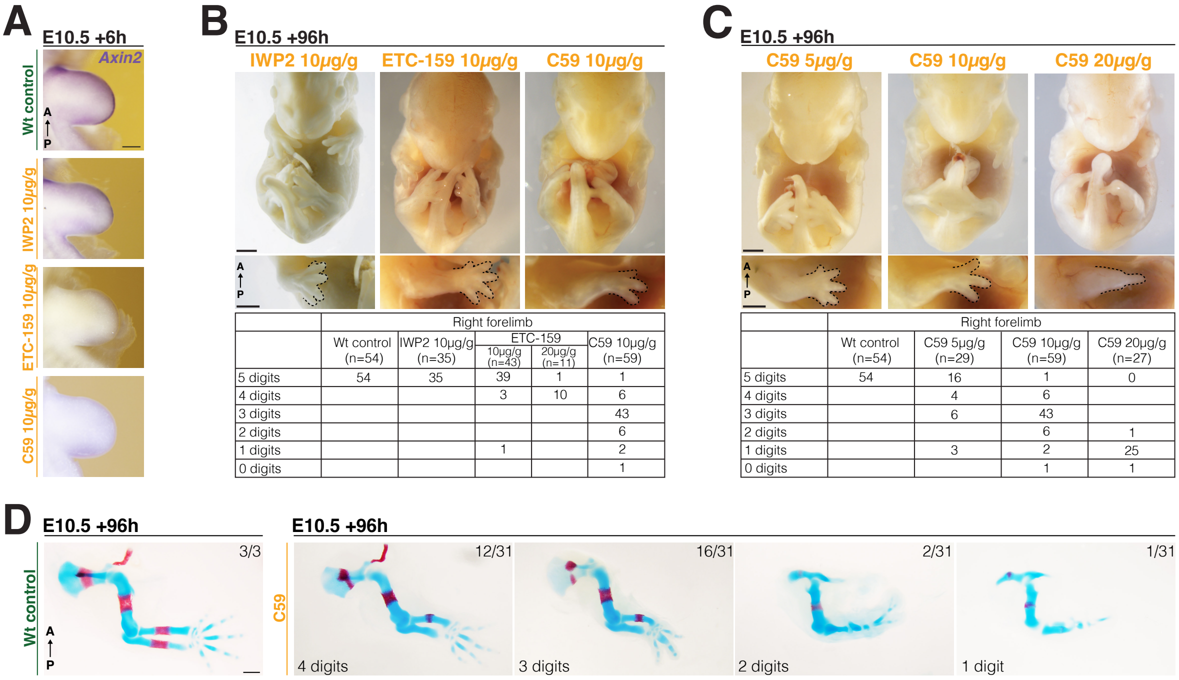

	Right forelimb				
	Wt control (n=54)	IWP2 10μg/g (n=35)	ETC-159 10μg/g (n=43)	ETC-159 20μg/g (n=11)	C59 10μg/g (n=59)
5 digits	54	35	39	1	1
4 digits			3	10	6
3 digits					43
2 digits					6
1 digits			1		2
0 digits					1

	Right forelimb			
	Wt control (n=54)	C59 5μg/g (n=29)	C59 10μg/g (n=59)	C59 20μg/g (n=27)
5 digits	54	16	1	0
4 digits		4	6	
3 digits		6	43	
2 digits			6	1
1 digits		3	2	25
0 digits			1	1

Fig. S3. IP injection of small molecule inhibitors identifies Wnt-C59 as the most suited WNT signaling inhibitor in mouse embryos. (A-C) Pilot study to test the effects on *Axin2* expression (panel A), limb skeletal and digit development (panels B, C) following IP injection of a single dose of 10μg per gram (10μg/g) body weight of either IWP2, ETC-159 and Wnt-C59 at embryonic day E10.5. **(A)** After 6hrs, the WNT transcriptional target *Axin2* is reduced but not lost from limb buds of IWP2-treated embryos (compared to wild-type controls). In contrast, *Axin2* expression is reduced/lost from limb buds of ETC-159 and C59-treated embryos (see also Fig. 1B for C-59). **(B)** The alterations of embryonic and forelimb skeletal development were assessed by morphological scoring. Upper and middle panels show representative whole embryos and forelimb buds for each of the three inhibitors (10μg/g) at +96hrs after IP injection (~E14.5). The table below lists the distributions of digit alterations observed. IWP2 does not alter digit development in agreement with incomplete loss of *Axin2* expression (panel A). IP injection of ETC-159 (10μg/g) causes low frequency digit loss (4/43). The frequency of tetradactyly is increased by IP injection of 20μg/g ETC-159 (10/11, see table). In contrast, morphological scoring of C59-treated embryos reveals robust digit alterations, most commonly reductions to 3 (43/54). **(C)** Dose-response analysis established 10μg/g C59 as the optimal concentration to induce oligodactylies in the

Development • Supplementary information

majority of all embryos, while 20µg/g C59 disrupts the periodic digit pattern (26/27). Scale bars: panel A 250*µ*m; panels B, C 1mm. **(D)** Definitive forelimb skeletal analysis shows that 10µg/g C59 causes predominant forelimb oligodactylies resulting in 4 digits (39%) or 3 digits (52%). These digit phenotypes are consistent with rapid but transient disruption of WNT signaling (see results section). All limbs are oriented with anterior to the top and posterior to the bottom. Scale bar: 500µm.

Development • Supplementary information

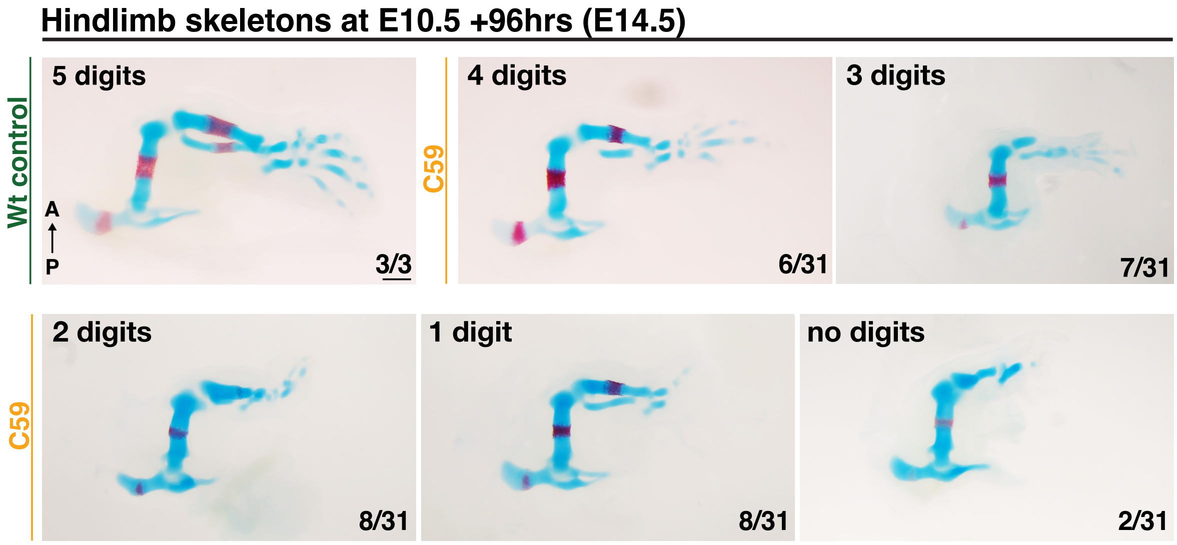

Fig. S4. Hindlimb skeletal analysis (E14.5) of embryos treated with 10µg/g C59 at E10.5. The fractions of limb skeletons with particular digit losses are indicated. All limbs are oriented with anterior to the top and posterior to the bottom. Scale bar: 500µm.

Development • Supplementary information

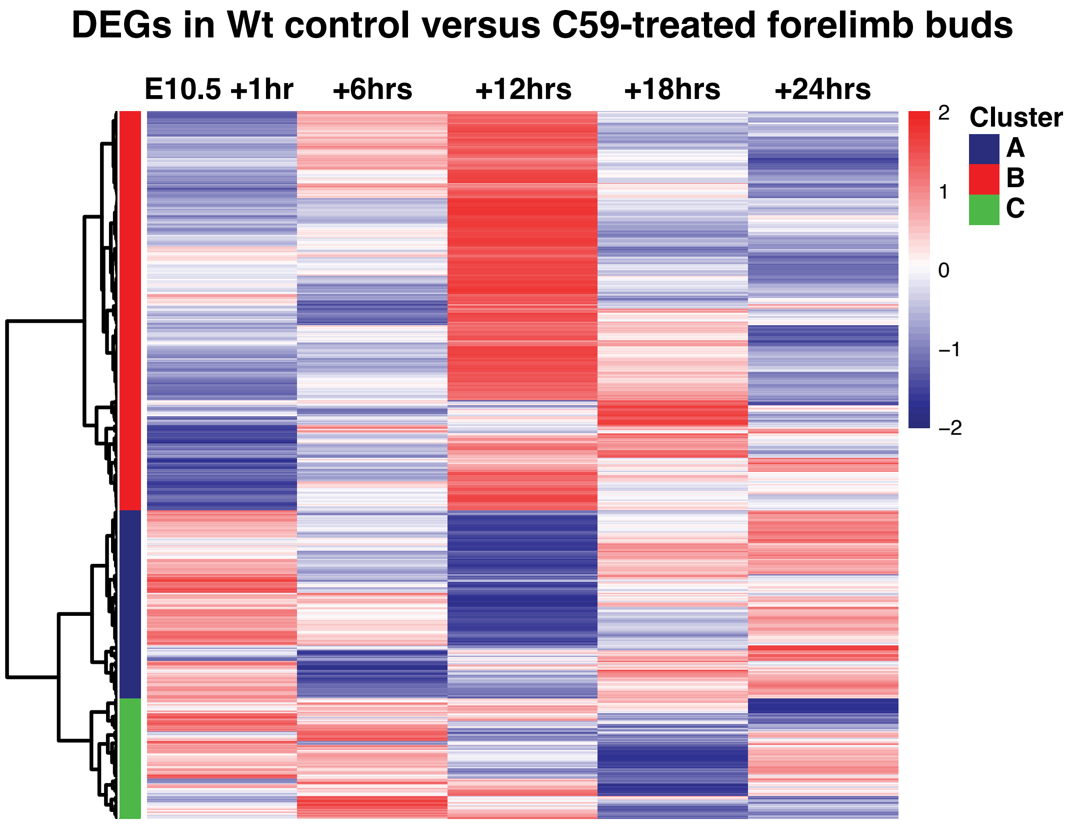

Fig. S5. Time course RNA-seq analysis of wild-type control and C59-treated forelimb buds. Heatmap of all DEGs shared between the replicates were clustered according to their expression profiles over time. The heatmap shows the log2FC centred and scaled clustering by correlation. All genes shown are significantly changed at minimally one timepoint in wild-type controls versus C59-treated forelimb buds. DEGs with downregulated (blue) and upregulated (red) expression in forelimb buds following C59-treatments are shown.

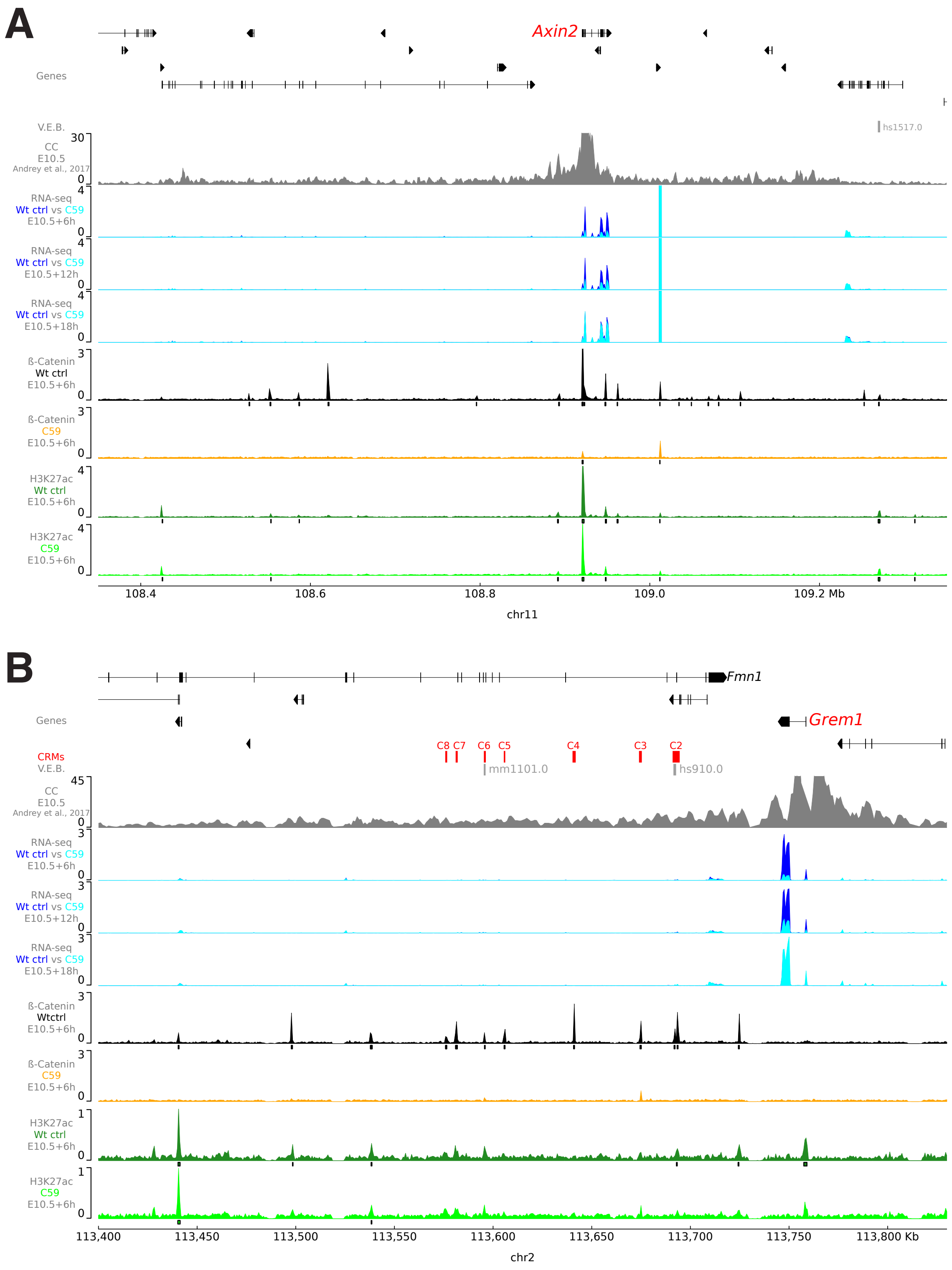
A
Axin2
Genes
V.E.B.
hs1517.0
CC
E10.5
Andrey et al., 2017
RNA-seq
Wt ctrl vs C59
E10.5+6h
E10.5+12h
E10.5+18h
ß-Catenin
Wt ctrl
C59
H3K27ac
108.4
108.6
108.8
109.0
109.2 Mb
chr11
B
Fmn1
Grem1
CRMs
C8 C7 C6 C5 C4 C3 C2
mm1101.0
hs910.0
Wtctrl
113,400
113,450
113,500
113,550
113,600
113,650
113,700
113,750
113,800 Kb
chr2

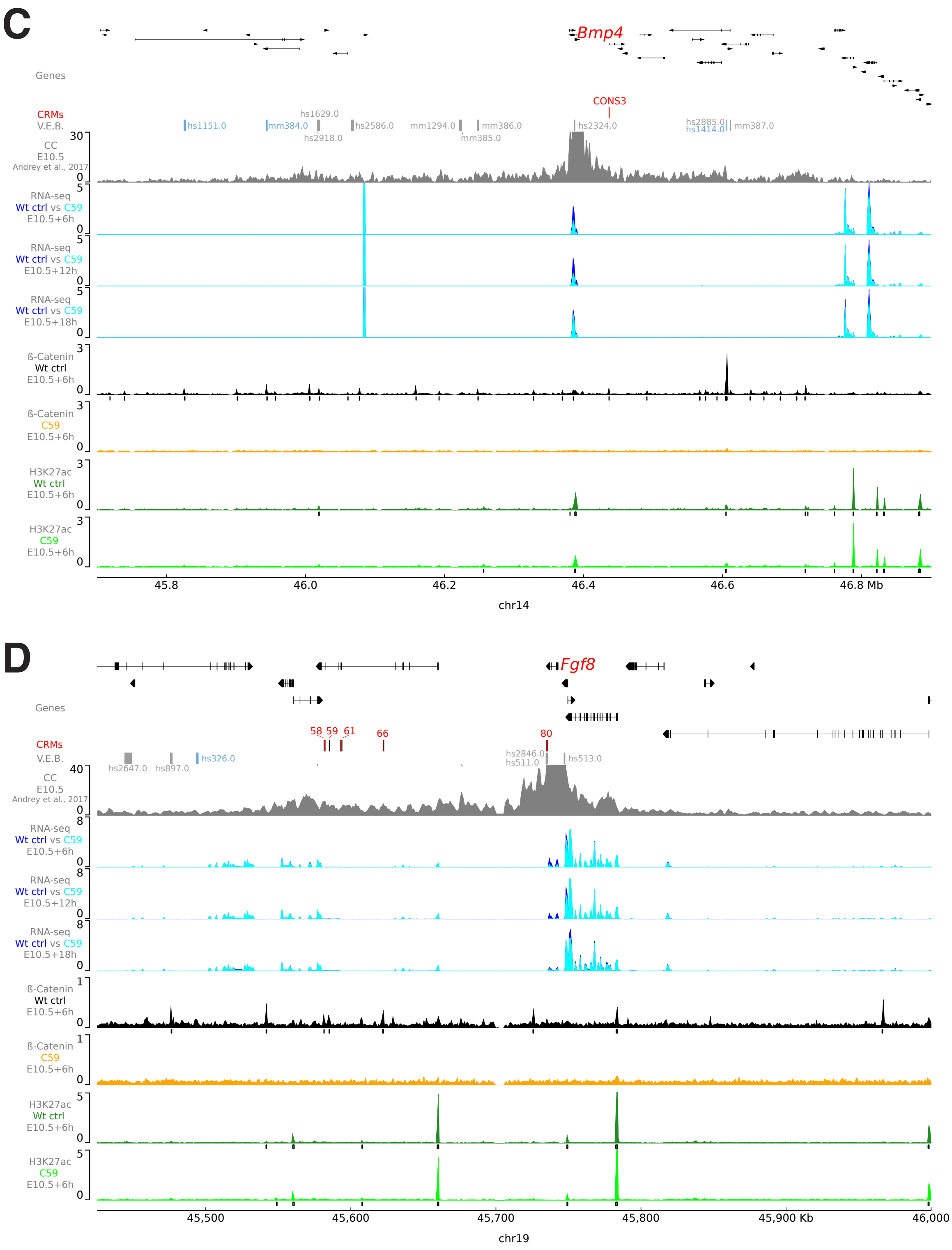

Development • Supplementary information

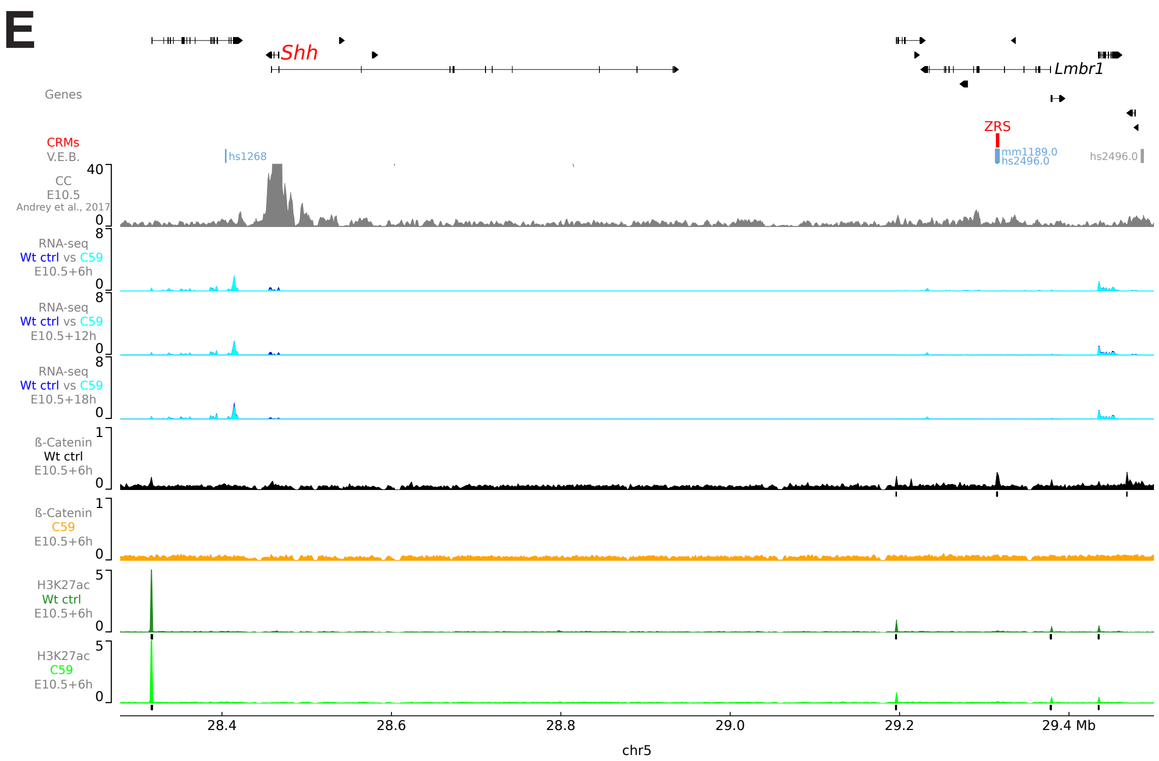

Fig. S6. C59 disrupts β-catenin interactions with CRM enhancers in genomic landscapes of DEGs following IP injection at E10.5 +6hrs. These graphs show additional information for the *Axin2*, *Grem1*, *Bmp4* and *Fgf8* gene regulatory landscapes (Fig. 2E-H). In addition the *Shh* genomic landscape is shown in panel E. (A-E) Previously identified CRMs (red) and tested regulatory elements. V.E.B: https://enhancer.lbl.gov/vista/ Click or tap here to enter text.; blue: active in limb buds, grey: no activity in limb buds). The regions that likely function as *cis*-regulatory landscapes for the DEGs of interest were approximated by plotting promoter-capture profiles (tracks CC, E10.5). In addition, RNAseq profiles of C59-treated (cyan) forelimb buds were plotted over the wild-type control profiles (Wt Ctrl, blue) to reveal the alterations in expression levels at E10.5+6, E10.5+12 and E10.5+18 hours. The binding of β-catenin chromatin complexes to the relevant genomic regions is shown for wild-type control (black) and C59-treated (orange) forelimb buds at E10.5+6hrs. This analysis indicates that the depletion of β-catenin from known and candidate CRMs rapidly alters expression of the target gene (indicated in red). The histone H3K27Ac profiles are shown for wild-type control (green) and C59-treated (lime) forelimb buds at E10.5 +6hrs. Note that only few differences are observed between wild-type and C59-treated limb buds. Called ChIPseq peaks are indicated by black bars below tracks.

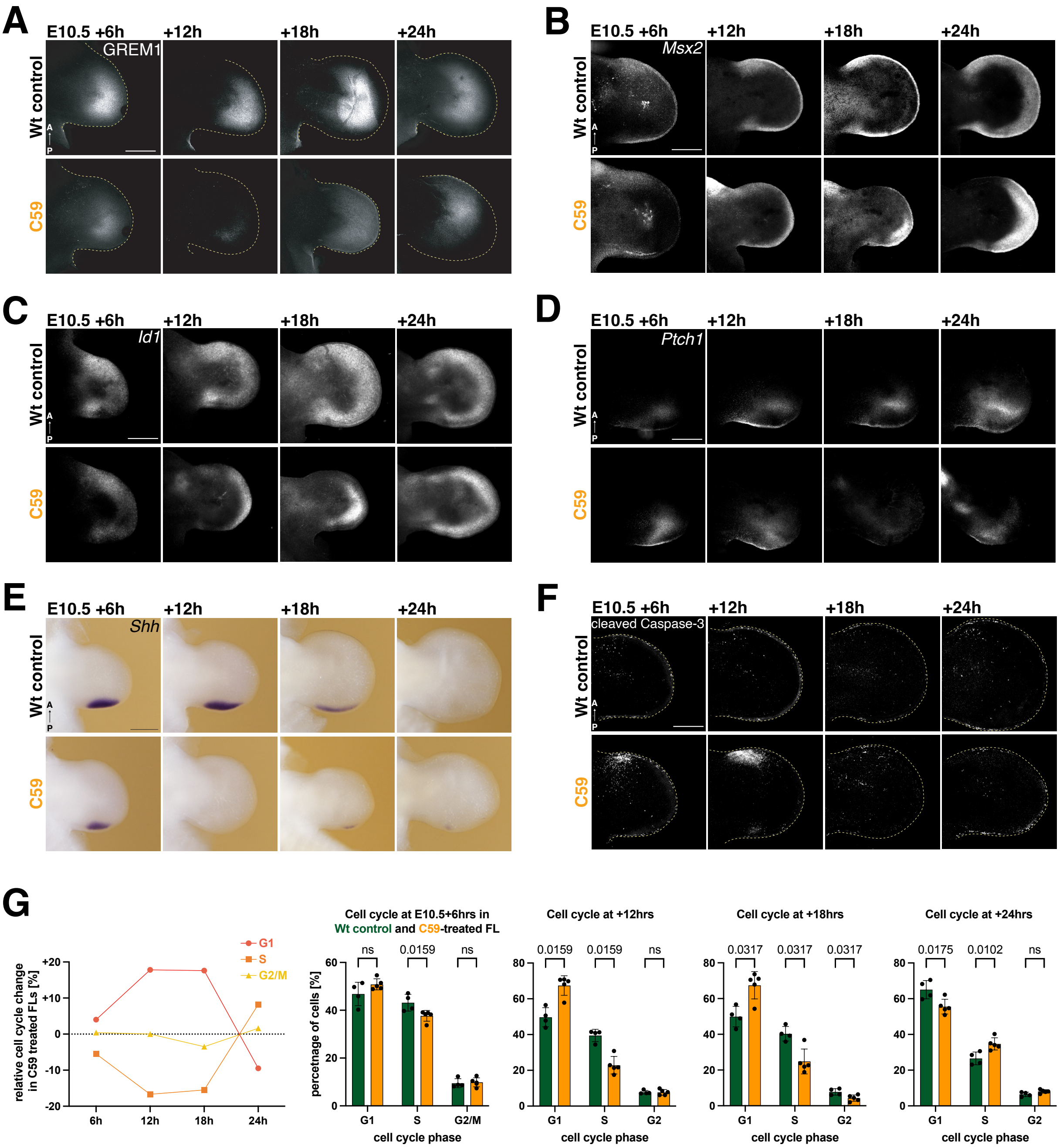

Fig. S7. Spatial expression kinetics of BMP and SHH target genes, transient apoptosis and cell cycle alterations following C59 treatment at ~E10.5. Spatio-temporal gene expression was assessed in wild-type control (upper panels) and C59-treated forelimb buds (lower panels) during disruption (E10.5 +6, +12hrs) and recovery of WNT-signaling (E10.5 +18, +24hrs). **(A)** The spatio-temporal distribution of the GREM1-protein was analysed by whole mount immunofluorescence. This reveals the

Development • Supplementary information

rapid clearance and re-expression of GREM1 during inhibition and restoration of WNT signaling. The temporal kinetics of GREM1 loss and restoration closely follow the alterations in *Grem1* transcription (Figs. 3A, 4C). **(B)** Rapid downregulation of the BMP target gene *Msx2* (E10.5+6hrs) is followed by recovery (E10.5+18-24hrs) in C59-treated forelimb buds. **(C)** Expression of the BMP target gene *Id1* in response to C59-mediated WNT inhibition is reduced and distalized by E10.5+12hrs and recovers parallel to WNT signaling. **(D)** Expression of the SHH transcriptional target *Ptch1* is spatially reduced by E10.5+18hrs after C59-treatment. **(E)** Conventional WISH analysis of the spatio-temporal kinetics of *Shh* expression. n=3 biological replicates were analysed per gene and stage. **(F)** Apoptotic cells are detected by cleaved Caspase-3 using immunofluorescence. Transient apoptosis is detected in the anterior mesenchyme of C59-treated forelimb buds at E10.5 +6 and +12hrs (n=3/3). Images are shown as maximum intensity projection of the entire or selected Z-stack range. Forelimb buds are oriented with anterior to the top and posterior to the bottom. Scale bars 200µm. **(G)** Limb bud cell proliferation was assessed by DAPI staining of DNA that was followed by flowcytometric analysis. The relative cell cycle changes over time are shown as a line plot (left-most panel). The fraction of cells in the G1, S and G2/M phases of the cell cycle in wild-type (green) and C59-treated limb buds (orange) are shown as bar plots. Statistical analysis was done using the Mann-Whitney test. p-values ≤0.05 are indicated. ns: not significant.

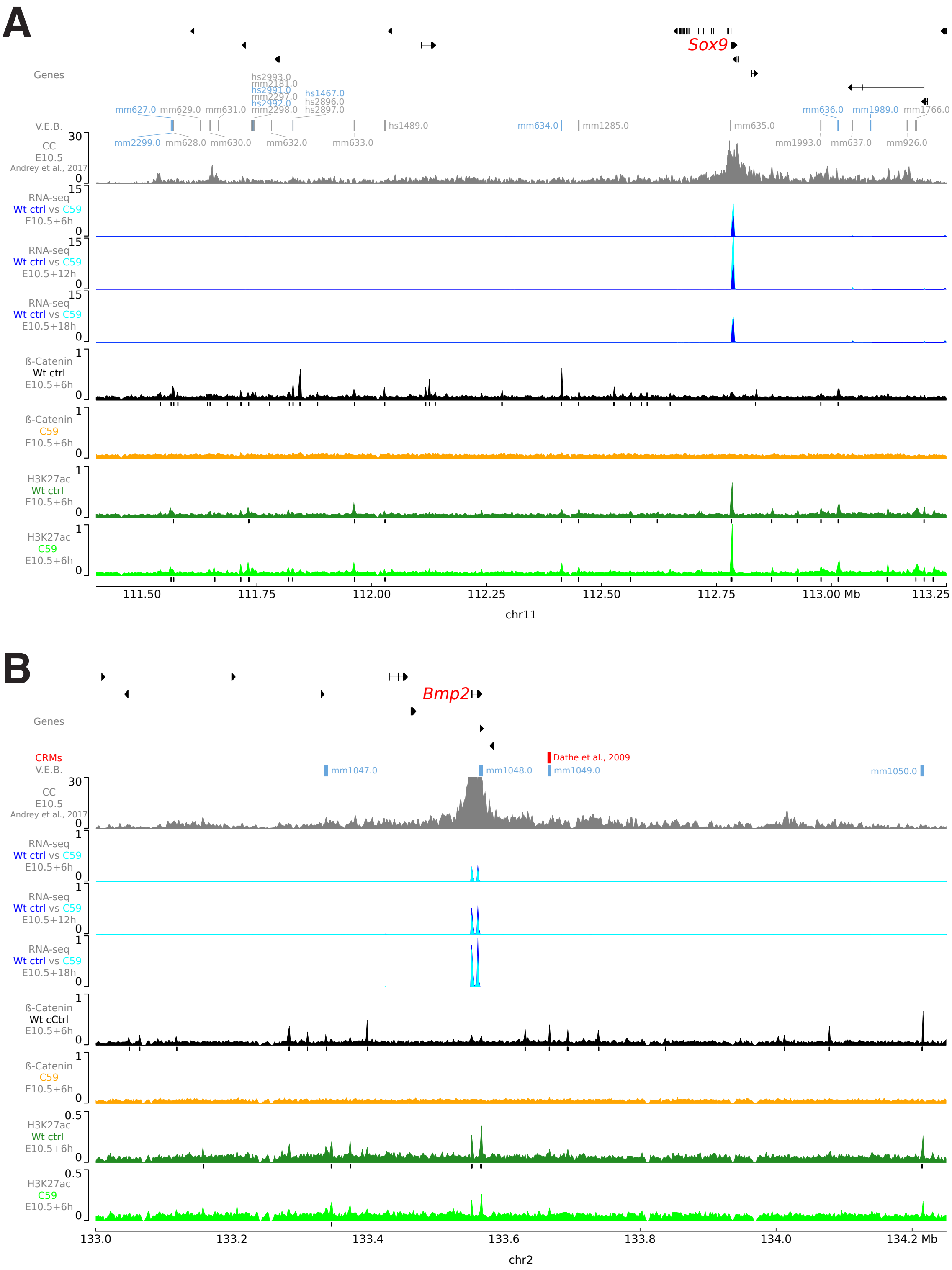

Development • Supplementary information

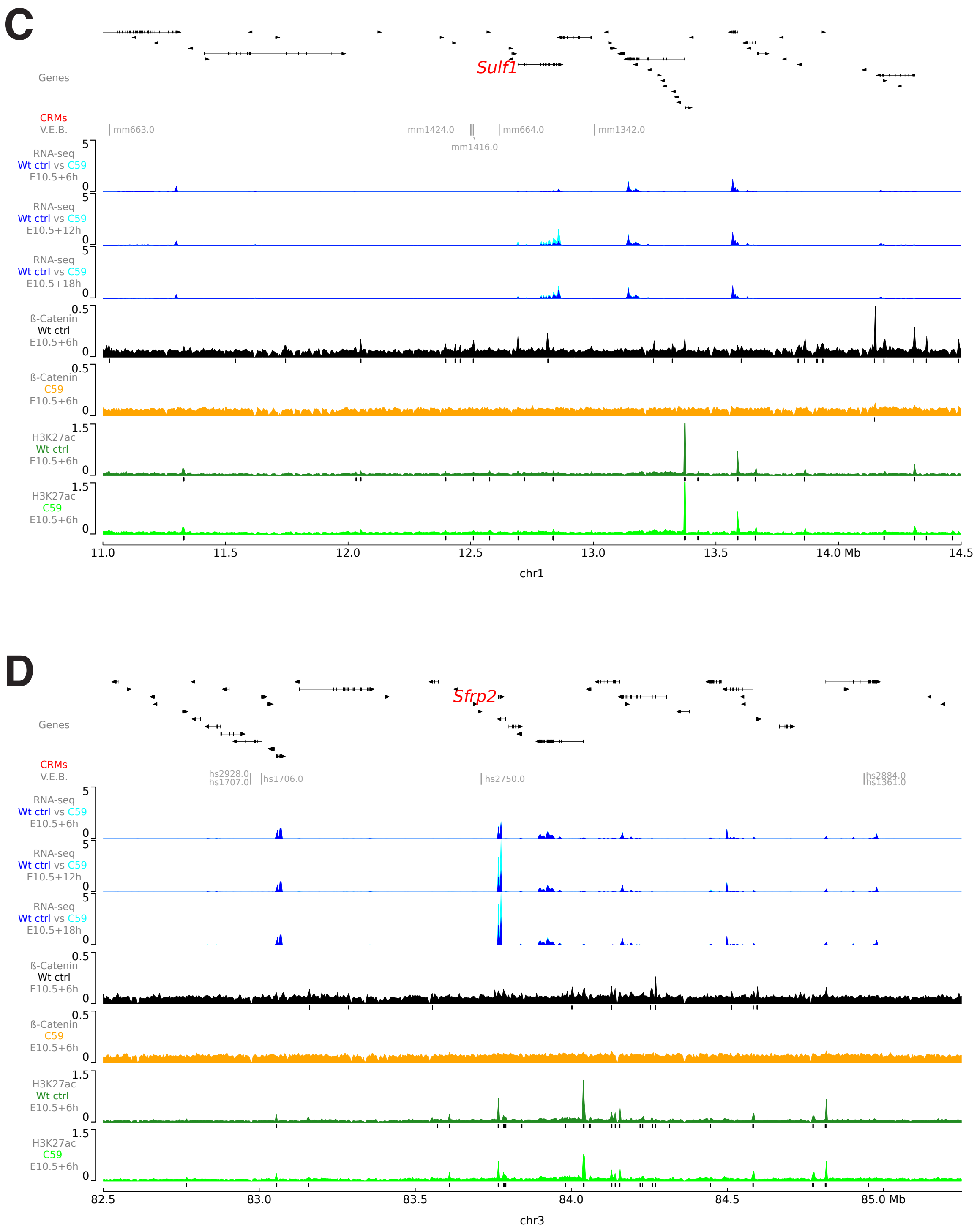

Fig. S8. β-catenin binding to regions in genomic landscape responding to disruption of WNT signaling by C59 IP injection at E10.5 +6hrs. Genomic landscapes of *Sox9* **(A)**, which is negatively regulated by WNT/β-catenin (i.e. expanded in C59-treated limb buds); *Bmp2* **(B)** which is positively regulated by WNT/β-catenin; *Sulf1* **(C)** and *Sfpr2* **(D)**, which are restricted by WNT signaling (i.e. expanded in C59-treated limb buds). **(A-D)** Previously identified CRMs (red) and tested regulatory

Development • Supplementary information

elements (V.E.B: https://enhancer.lbl.gov/vista/, blue: active in limb buds, grey: no limb bud activity). The regions that likely function as *cis*-regulatory landscapes for these DEGs were approximated by plotting promoter-capture profiles (tracks CC, E10.5, not available for *Sulf1* and *Sfrp2,* Andrey 2017). In addition, RNAseq profiles of C59-treated (cyan) forelimb buds were plotted over wild-type control profiles (Wt Ctrl, blue) to reveal the alterations in expression levels at E10.5 +6, +12 and +18 hrs. The interactions of β-catenin chromatin complexes within the genomic landscapes is shown for wild-type control (black) and C59-treated (orange) forelimb buds at E10.5 +6hrs. Together this analysis indicates that the depletion of β-catenin chromatin complexes from CRMs underlies the transcriptional alteration of the associated DEG (indicated in red). The histone modification H3K27Ac profiles are shown for wild-type control (green) and C59-treated (lime) forelimb buds at E10.5 +6hrs. Note that only few differences are observed between wild-type and C59-treated limb buds. Called ChIPseq peaks are indicated by black bars below tracks.

Development • Supplementary information

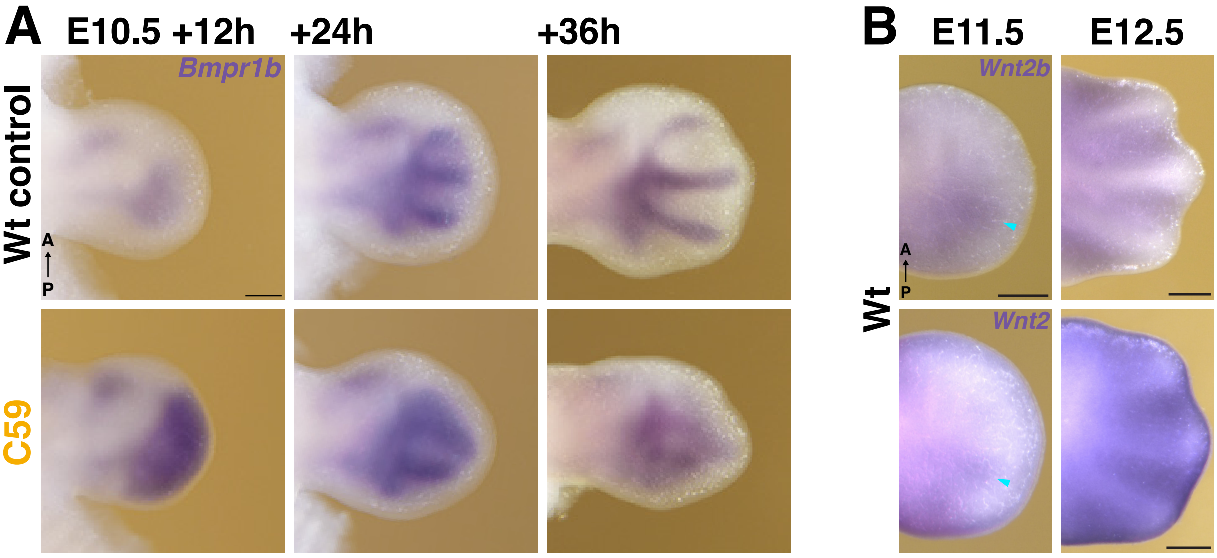

Fig. S9. Altered *Bmpr1b* expression dynamics in C59-treated forelimb buds and the spatial expression of *Wnt2* and *Wnt2b* in wild-type forelimb buds. (A) Spatial dynamics of the *Bmpr1b* receptor expression during disruption and recovery of WNT signaling. **(B)** *Wnt2b* (top panels) and *Wnt2* ligand expression (bottom panels) in the presumptive interdigit mesenchyme of wild-type forelimb buds at E11.5 and E12.5. Scalebars: 250μm. A=anterior, P=posterior. n=3 biological replicates were analysed for all timepoints and genes expression patterns shown.

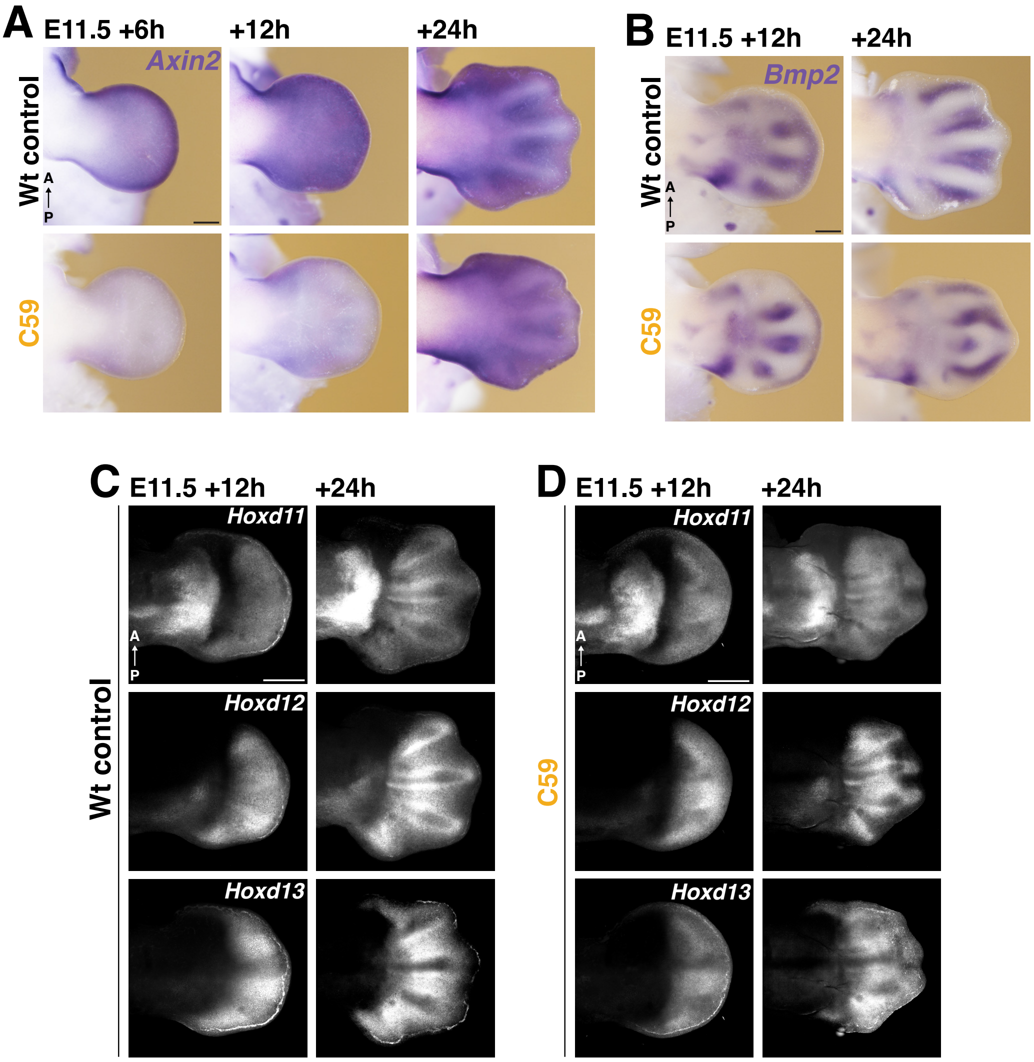

Fig. S10. Temporal alterations and recovery of the periodic digit-interdigit patterning system after transiently disrupting WNT signaling at ~E11.5. (A) *Axin2* expression in wild-type control and C59-treated forelimb buds at E11.5 +6, +12 and +24hrs. This establishes that C59-mediated inhibition and recovery of WNT signaling occurs with kinetics similar to inhibition at ~E10.5. **(B)** Spatio-temporal *Bmp2* expression at E11.5 +12 and +24hrs wild-type control and C59-treated forelimb buds. **(C, D)** Greyscale single channels for the analysis shown in Fig. 6F. *Hoxd11*, *Hoxd12* and *Hoxd13* expression in wild-type control (panel C) and C59-treated forelimb buds (panel D). n=3 biological replicates per probe and stage were analysed. All limb buds are shown with anterior to the top and posterior to the bottom. Scale bars in panel A-C: 250µm; panel D, E: 200µm.

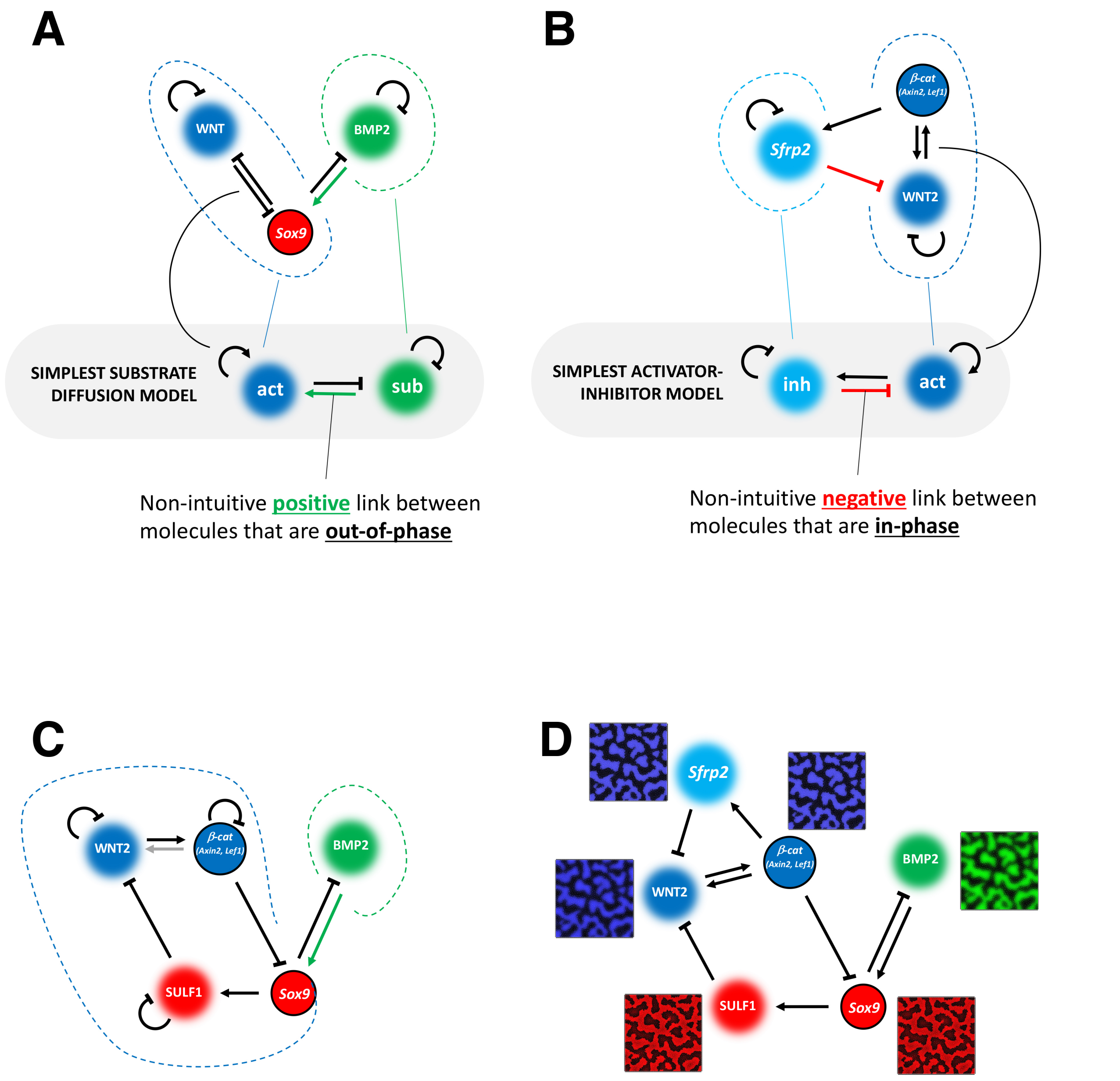

Fig. S11. Scheme to illustrate how classical Turing models (substrate-depletion and activator-inhibitor) map into the extended new circuit hypothesis. (A) In the original BSW Turing model, the substrate is represented by BMP2, while the activator is represented by the interaction between Sox9 and WNT. Auto-activation of the activator is achieved by mutual-repression between Sox9 and WNT. The non-intuitive aspect of the substrate-depletion model is the green positive arrow: the substrate has a positive effect on the activator, despite the two molecules being out-of-phase. For

Development • Supplementary information

the BSW Turing model this explains how BMP2 can be out-of-phase with Sox9 as *Bmp2* is expressed by the prospective interdigit mesenchyme, while *Sox9* is expressed by prospective digit mesenchyme. Despite this, BMP2 has a positive regulatory effect on *Sox9*. **(B)** The 3-node model shown in Fig. 7B can be explained as an activator-inhibitor model, in which Sfrp2 acts as the inhibitor, while the activator is represented by a positive feedback loop between WNT2 and β-catenin. The non-intuitive aspect of an activator-inhibitor model is that the inhibitor represses the activator (red link) but both molecules are active in the same tissue (in-phase). **(C)** The extended circuit hypothesis presented in Fig. 7D can also be explained as a substrate-depletion model (compare panels C and A). **(D)** The full hypothesis (6-node circuit) in Fig. 7E was simulated in a 2D spatial domain, which confirms that all species are expressed in the expected phases with respect to each other (insets).

Table S1. Temporal profile of differentially expressed genes. DEGs identified at 1, 6, 12, 18 and 24hrs after C59 injection. Linked to Fig. 1, 2A.

Available for download at
https://journals.biologists.com/dev/article-lookup/doi/10.1242/dev.204606#supplementary-data

Table S2. DEG fold-change expression profiles over time. DEGs were split into three clusters based on their temporal fold-change expression profiles. Linked to Fig. 1D.

Available for download at
https://journals.biologists.com/dev/article-lookup/doi/10.1242/dev.204606#supplementary-data

Table S3. DEGs selected for line plots analysis of the relevant pathways. Linked to Fig 1E.

Available for download at
https://journals.biologists.com/dev/article-lookup/doi/10.1242/dev.204606#supplementary-data

Table S4. DEGs at the E10.5 +6hrs timepoint selected for network analysis. Linked to Fig. 2A.

Available for download at
https://journals.biologists.com/dev/article-lookup/doi/10.1242/dev.204606#supplementary-data
