# Supplementary material for: WNT signaling coordinately controls mouse limb bud outgrowth and establishment of the digit-interdigit pattern
Source: Development. 2025 Jun 10;152(11):dev204606. doi: 10.1242/dev.204606 (PMC12188246; doi:10.1242/dev.204606)
Supplement: Supplementary file 1 [file develop-152-204606_review_history.pdf]

WNT signaling coordinately controls mouse limb bud outgrowth and establishment of the digit-interdigit pattern

Jonas Malkmus, Angela Morabito, Lucille Lopez-Delisle, Laura Avino-Esteban, Alexandre Mayran, Aimee Zuniga, James Sharpe, Rolf Zeller and Rushikesch Sheth
DOI: 10.1242/dev.204606

Editor: Liz Robertson

Review timeline

Original submission:	20 December 2024
Editorial decision:	20 January 2025
First revision received:	16 April 2025
Accepted:	6 May 2025

Original submission

First decision letter

MS ID#: dev.204606

MS Title: WNT signaling coordinately controls mouse limb bud outgrowth and establishment of the digit-interdigit pattern

Authors: Rolf Zeller; Jonas Malkmus; Angela Morabito; Lucille Lopez-Delisle; Laura Avino Esteban; Alexandre Mayran; Aimee Zuniga; James Sharpe; Rushikesch Sheth
Article Type: Research Article

Dear Dr Zeller,

I have now received all the referees reports on the above manuscript, and have reached a decision. The referees' comments are appended below, or you can access them online: please go to.

The overall evaluation is positive and we would like to publish a revised manuscript in Development, provided that the referees' comments can be satisfactorily addressed. Please attend to all of the reviewers' comments in your revised manuscript and detail them in your point-by-point response. If you do not agree with any of their criticisms or suggestions explain clearly why this is so. If it would be helpful, you are welcome to contact us to discuss your revision in greater detail. Please send us a point-by-point response indicating your plans for addressing the referees' comments, and we will look over this and provide further guidance.

Reviewer 1

SUMMARY OF THE ADVANCE MADE IN THIS PAPER AND ITS POTENTIAL SIGNIFICANCE TO THE FIELD

WNT signalling is involved in many developmental scenarios, including those that operate to form the limb. This manuscript is a valuable contribution to understanding limb development, and pattern formation more generally, through its interrogation of the molecular and morphological effects of timed suppression of WNT signalling, and its interpretation of results in the frame of developmental models. The data are convincing and novel and the work advances understanding of the Turing-type process that appears to operate to define digit periodicity, identifying new interactions and molecules likely to act in this system. The reported recovery of limb morphology

© 2025. Published by The Company of Biologists under the terms of the Creative Commons Attribution License (https://creativecommons.org/licenses/by/4.0/).

from transient suppression of WNT production is interesting and relevant to understanding developmental robustness, and also to teratology and the effects of environmental perturbation on morphology and congenital conditions. The experimentation presented is extensive and rigorous and I have only some corrections and minor suggestions, which the authors may wish to consider in a revision.

SUGGESTIONS TO AUTHORS

The 'biological n' should be defined in the methods. Is it an individual embryo or a litter/treated pregnant female?

It is interesting and I think worth noting that the rapidly detectable effect of C59 administration on gene expression implies that the pool of extracellular WNT proteins in this system is short lived and turning over very quickly. This supports the idea that it might act in a highly dynamic regulatory system (like the Turing-type system proposed).

A potential mechanism underlying the recovery of limb morphology following temporary WNT inhibition is given (poised multiprotein complexes at enhancers) but a little more might be said here. More generally, it seems like the question is whether there is a persistent molecular state (long-lived protein complexes at enhancers could be one, though, for example, spatially discrete long-lived mRNAs for factors not considered here, or long-lived cytoplasmic proteins could be others) that might explain this recovery phenomenon, or whether the patterning system itself reverts to a corrected state and so the developmental process is at least somewhat path independent. For example, the final pattern could be one that would be produced on the geometry of the autopod field if the Turing system worked only at a late stage, and not rely on its steady action and emergence of form over the past hours/days.

It would be worthwhile to explain the systems presented in figure 7 some more. With more components and interactions they become harder to understand at an intuitive level. For the substrate-depletion element (Fig 7D), comparing this to a simple two component substrate-depletion model there should be some freely-diffusing component that is present in limiting concentration and some other component that depletes it (as SULF1 does to WNT2), but the depleting factor should have limited diffusion and be self-enhancing? Which component(s) achieve these criteria or functions? If WNT is the substrate then it must diffuse very readily and this is worth noting as a constraint, and then SULF1 achieves self-enhancement through suppressing WNT, which represses Sox9, which activates SULF1. For Fig 7B, the activator inhibitor system, however, WNT would seem to need to undergo more constrained diffusion, at least diffusing less than Sfrp2, if the latter is the inhibitor. Then in 7E the degree to which this system should be regarded as activator-inhibitor or substrate-depletion could depend on the diffusivity of the WNT and how much WNT signal is blocked by SULF1 (S-D) compared to how much is blocked by Sfrp2 (A-I). BMP seems like it must diffuse readily in any of these models if it is to stimulate Sox9 expression in the digital mesenchyme. I realise it is difficult to know where to stop with these explanations as they can become very wordy but a little more guidance for the reader on any key constraints or properties of the models, such as relative diffusion rates of species, and how these vary between the models presented, would be helpful.

Related to the models, are there really only two relevant expression domains (digit and interdigit) for the system? Fig 6D, for example, suggests that there might be more than this. In addition, Sulf1appears to extend more distally than Sfrp2 - is Sulf1 expression activated earlier than Sfrp2's and would this be relevant to the model?

In the text related to Fig 7 (p23) it is stated that SULF1 positively modulates BMP signalling, but in Fig 7E SULF1 has an inhibitory effect on BMP. Which is correct?

Fig 3A would benefit from annotation with arrows or similar to show the different genes expression domains listed (since they are all the same colour ISH signal)

In Fig 7 legend specify what the grey versus black interaction lines signify

© 2025. Published by The Company of Biologists under the terms of the Creative Commons Attribution License (https://creativecommons.org/licenses/by/4.0/).

In Fig S6B the Msx1expression at 6h looks stronger than in the control condition. Can the authors explain this?

Corrections:

p14 'constraint' --> 'constrained'

p15 'wild type' would be better as 'control' or similar s genotypes are not different between conditions here

p19 'WNT pathway is' --> 'as'

Fig S1 legend title has an extra '7'

Fig S2F should have a Sox9 label on the panel

The figures for Fig S3 and Fig S4 have been swapped and so sit above the wrong legend

Wnt2a is occasionally mentioned in the text but I think this is the same gene is more commonly referred to in the text as Wnt2. If there are three relevant Wnts (Wnt2, Wnt2a, Wnt2b) then please explain, if there are only two relevant Wnts (Wnt2=Wnt2a, Wnt2b) then please choose one designation and use that only throughout

Reviewer 2

In this study, Malkmus et al. address the consequences of acute and transient Wnt signaling inhibition on mouse forelimb digit patterning, and identify the Wnt pathway at the core of a self-regulating Turing system. They employ the small molecule C59, which blocks the secretion of all WNT ligands, to transiently arrest Wnt signaling in embryos at 10.5 dpc during the early phases of forelimb bud development. Doing so allows expansion of the digit-specifying territory, marked by broadened expression of the cartilage master gene Sox9. Wnt signaling recovery at 36h post injection is sufficient to re-establish a self-regulatory system where digit and interdigit territories are marked, yet with delayed specification causing the reduction of digit number. The data foster a model whereby it is not only positional information driven by SHH - the most widely held view - but rather a complex network of feedback loops (similar to a Turing system) capable of self-regulating and resulting in repetitive digit-interdigit territories. The question underlying the study is important, and the methods employed to respond it elegant and persuasive.

Here I present a series of comments that, if taken into consideration, have the potential to improve, in my opinion, the impact and the quality of the article.

1) Three different inhibitors are compared and C59 is chosen based on the severity of phenotype. The study is therefore entirely based on using this compound. Formally, I would have expected that the choice of which inhibitor to use should be made based on a Wnt-specificity readout, for example by measuring which one leads to the strongest downregulation of Axin2, while basing this decision on the severity of phenotype induced might be seen as cherry-picking. Axin2 reduction is only shown for C59 (Fig. 1B). While the effects on gene regulatory networks measured in the rest of the study do point to a specific effect, I am left wondering why the other inhibitors did not cause any (or much) phenotype. Have the authors assessed Axin2 expression upon their injection? This would help to sort this out: the absence on effects on Axin2 would suggest that other inhibitors simply do not work in this context. If, on the other hand, IWP2 and ETC-59 caused Axin2 downregulation but no phenotype, this might indicate a possible toxicity/off-target consequences induced by C59, which would warrant additional controls.

2) Throughout the study the authors looked at forelimbs, and do not show data on hindlimbs (apart from something that could be glimpsed in Fig. S4). As the latter are slightly delayed during development, this seems like the perfect chance of manipulating Wnt signaling in the two similar

© 2025. Published by The Company of Biologists under the terms of the Creative Commons Attribution License (https://creativecommons.org/licenses/by/4.0/).

but not identical system in one go. If the authors have any image or data on the hindlimbs' phenotype across their experiments, I encourage them to include these in the study.

3) Sox9 expands into the sub-ectodermal mesenchyme upon beta-catenin deletion (S2D). What has made the authors exclude that, instead of an expansion of expression, sub-ectodermal and ectodermal cells are lost? After all, the surface ectoderm is an epithelium susceptible to disintegration without beta-catenin adhesive function, irrespectively of Wnt signaling activation.

4) I truly appreciated the molecular data showing beta-catenin genomic occupancy by ChIP-seq, and how beta-catenin peaks vanish with the use of the inhibitor whilst H3K27ac remains unaffected. This is truly compelling. One comment on the signal profile in Figure 2B: the profiles of H3K27ac in control and C59 look to me identical (I cannot spot any pixel difference between the two). Normally, two ChIP-seq replicates of even the same experiment yield slight, even if minimal, differences. Could the authors make sure that no image was used twice by mistake? I also recommend showing the H3K27ac peaks (not only the signal profile): these would be nice to see in the main figure*, but if the authors do not like this option, they are also ok as supplementary dataset. *Unperturbed H3K27ac, in fact, while interesting per se as it indicates that chromatin conformation is not perturbed in these loci in the absence of beta-catenin, is also an important control that the experiment in C59 injected embryos worked, landing credibility that the disappearance of beta-catenin from these positions is specific and not a consequence of e.g. cell death.

5) Concerning the ChIP-seq on beta-catenin, the authors emphasize positively regulated direct targets. Yet their analysis point to considerable repressive action of Wnt signaling on the cartilage program. We have also seen a potentially direct β-catenin-mediated downregulation, a phenomenon unexplained in the field (PMID: 37473729). Could the authors find beta-catenin-bound loci that match downregulated genes in their proximity? Interesting is for example the difference in behaviour between the adjacent Hoxd12 and Hoxd13.

Minor points:

6) I find the use of the word "sensor" for target genes misleading. Reporter would be, in my opinion, better, as Axin2 is used as a proxy for the Wnt signaling status, while sensor implies the generation of an output based on the detection of a signal over a threshold.

7) At page 8, reference to Fig. S3 in describing the three inhibitors should be to Fig. S4 instead.

8) In figure S2F the legend "Sox9" in the image is missing.

9) At page 7, up in the paragraph there is a typo "during in": one of the two words should be deleted.

10) At page 11, the reference to Fig. 2A on Grem1/Bmp peaks should be to Fig. 2G/2F.

11) On page 14, "which indicates that the interdigit is a source for WNT ligand(s) functioning in digit-interdigit patterning (Fig. 4D)", perhaps the reference should be to 4E?

12) In Figure 3A it is relatively straightforward to distinguish the three classic markers, for someone in the field, based on their position. Yet, it is not clear to me why the authors use a grayscale where one could not specifically distinguish Shh downregulation.

13) The authors suggest that Wnt targets might include Bmp2 (Figure 4B). We have previously found and focused on precisely this (see figure 11 of PMID: 30735122). Do the authors find the same peak within the Bmp2 locus in their β-catenin ChIP-seq? If so, the results align and are a good support of each other.

14) At page 6, the authors write "slow kinetics" and "disruptive nature" of genetic beta-catenin inactivation. I found these statement qualitative and weak as rationale to justify the use of C59. C59, in fact, inhibiting porcupine also has an arguably slow kinetics (compared to direct beta-catenin-TCF/LEF inhibitors, for instance).

© 2025. Published by The Company of Biologists under the terms of the Creative Commons Attribution License (https://creativecommons.org/licenses/by/4.0/).

Review by Claudio Cantú
(Why I sign: I believe that peer review should be a transparent dialogue. I strive to be critical but honest and professional, and care that my opinions and criticisms are formulated as if I were meeting the authors in person)

Reviewer 3

SUMMARY OF THE ADVANCE MADE IN THIS PAPER AND ITS POTENTIAL SIGNIFICANCE TO THE FIELD

It was already known that WNT and BMP signaling establish early limb bud and digit-interdigit patterning, however, the molecular transition between these two processes remained unexplored. Here, Malkmus et al., propose WNT signaling as the controlling signaling pathway in this transition. Initially, by conditional inactivation of beta-catenin they showed that WNT/betacat signaling is required for digit-interdigit and phalange patterning. Nevertheless, this inactivation was permanent and did not allow to distinguish between early and late WNT functions. By injecting a WNT small molecule inhibitor, C59, they circumvent this problem and rapidly and transiently abrogate WNT signaling. The abrogation and later recovery of WNT signaling is nicely shown by FISH-RNA and RNAseq experiments. It is also shown how this reduction results in rapid and transient reduction of genes involved in SHH, FGF and BMP pathways and upregulation of chondrogenic markers. In order to assess whether this regulation is direct, beta-catenin and H3K27ac ChIPseq experiments were performed. The analysis showed that most of the described and/or potential enhancers of the DEG genes overlap with beta-cat binding regions. These bindings are absent upon C39 treatment. Therefore, it is concluded that WNT directly regulate these genes by interaction of beta-cat with their enhancers.
It is also shown that there is a disruption in the digit-interdigit patterning establishment in C59-treated limbs and Sfrp2 and Sulf1 are identified as candidates for WNT modulation during this process.
To clarify WNT function at later time points during digit ray progression, C39 was injected at E11.5. Again, alterations in gene expression involved in early limb bud signaling and digit-interdigit patterning were observed which are restored upon WNT recovery with no impact on digit development.
The restoration of digit patterning once WNT is reestablished, both in the early (10.5) and late (E11.5) C59-treated limbs, indicates the plasticity and robustness of this process.

Overall, I found the manuscript to be well-written, with clear results and providing molecular elucidation in digit-interdigit domains establishment.

SUGGESTIONS TO AUTHORS

I have minor concerns about it. One of them is the lack of proliferation studies (more than just cluster C in figure 1D). Being WNT involved in the maintenance of limb mesoderm progenitors and given that the oligodactyly phenotype is caused by smaller handplates, a cell death/proliferation study is missing. Indeed, not just digits but all bones in stylopod and zeugopod are also smaller in the mutants (Fig4D). Although this WNT role is known it would improve the study describing the dynamics of proliferation in C59-treated limb buds.

While it is shown that genes involved in digit pattering are affected, some of these genes are also present and affected in the stylopod and zeugopod regions (for instance Sox9 in figure 4A). Moreover, RNAseq is done with the whole limb. This makes difficult to conclude any digit specific result at least from RNAseq experiments (p.9 last paragraph : "…while inhibiting precocious chondrogenic fates and upregulation of genes function in digit patterning ").

WNT non-canonical pathway has been shown to have a role in digit growth. How is the non-canonical pathway in your mutants? (RNAseq or FISH-RNA)

I miss the skeletal phenotype of Ctnnb1betac/betac

Figure legend of figure S3 and S4 are changed

© 2025. Published by The Company of Biologists under the terms of the Creative Commons Attribution License (https://creativecommons.org/licenses/by/4.0/).

Figure S3 (s4?) Time course RNA-seq analysis. Section A is almost not explained and it is not clear to me what authors want to show. Remove?

Minor:
p.7: during in (?)
p.8 Fig. S3 A,B does not correspond
p.23 into results (?)
4E figure legend. Colocalization?? Do they colocalize? Same in Fig.5C,D
Fig.S2F Sox9 labelling is missing

First revision

Author response to reviewers' comments

Reviewer's Comments

We are grateful to the 3 reviewers for the positive evaluation of our study and providing useful comments that we have all addressed by performing additional experiments, simulations and revising/extending the description in the Results, Discussion and Materials and Methods section and Main and Supplementary Figure legends. In addition several of the main and supplementary Figures are substantially revised. For reviewer's convenience we include a marked copy of the Manuscript text with main Figures (as PDF), in which all major changes are shown in red. In our opinion, these revision have significantly improved the study.

REVIEWER 1
WNT signalling is involved in many developmental scenarios, including those that operate to form the limb. This manuscript is a valuable contribution to understanding limb development, and pattern formation more generally, through its interrogation of the molecular and morphological effects of timed suppression of WNT signalling, and its interpretation of results in the frame of developmental models. The data are convincing and novel and the work advances understanding of the Turing-type process that appears to operate to define digit periodicity, identifying new interactions and molecules likely to act in this system. The reported recovery of limb morphology from transient suppression of WNT production is interesting and relevant to understanding developmental robustness, and also to teratology and the effects of environmental perturbation on morphology and congenital conditions. The experimentation presented is extensive and rigorous and I have only some corrections and minor suggestions, which the authors may wish to consider in a revision
We thank the reviewer for the positive evaluation of the study and very helpful comments and suggestions that we have addressed in revising the manuscript.

SUGGESTIONS TO AUTHORS

The 'biological n' should be defined in the methods. Is it an individual embryo or a litter/treated pregnant female?
We have modified the description of the biological replicates throughout the Materials and methods section:
For WISH, RNA-FISH, immunofluorescence and apoptosis analysis of wild-type and C59-treated forelimb buds: Biological replicates were generated by analysing forelimb buds from different DMSO or C59-treated-embryos. For each condition and time point the biological replicates included embryos from minimally three different litters at all timepoints. To avoid potential left-right bias, the limb buds analysed were chosen randomly.
For RNA-seq analysis: For each biological replicate, total RNA was isolated from two to three pairs of forelimb buds. No sex selection was done but genes on sex chromosomes were excluded from downstream analysis.
For ChIP-seq analysis: Per biological replicate 20 forelimb buds were pooled for analysis. For comparative cell cycle analysis of wildtype and C59-treated forelimb buds: one biological replicate

© 2025. Published by The Company of Biologists under the terms of the Creative Commons Attribution License (https://creativecommons.org/licenses/by/4.0/).

consists of forelimb buds of embryos from 1-2 litters (per treatment and timepoint).

A potential mechanism underlying the recovery of limb morphology following temporary WNT inhibition is given (poised multiprotein complexes at enhancers) but a little more might be said here. More generally, it seems like the question is whether there is a persistent molecular state (long-lived protein complexes at enhancers could be one, though, for example, spatially discrete long-lived mRNAs for factors not considered here, or long-lived cytoplasmic proteins could be others) that might explain this recovery phenomenon, or whether the patterning system itself reverts to a corrected state and so the developmental process is at least somewhat path independent. For example, the final pattern could be one that would be produced on the geometry of the autopod field if the Turing system worked only at a late stage, and not rely on its steady action and emergence of form over the past hours/days.
This is relevant to consider and we discuss this in some detail on page 20 (Discussion). As not much is known about the half-lives of transcriptional complexes or RNAs encoding other transcription factors in the context during of limb bud development, we prefer not to go into more speculation.

It is interesting and I think worth noting that the rapidly detectable effect of C59 administration on gene expression implies that the pool of extracellular WNT proteins in this system is short lived and turning over very quickly. This supports the idea that it might act in a highly dynamic regulatory system (like the Turing-type system proposed).
We briefly describe this on page 20 (Discussion) for WNT, but also for the BMP antagonist Grem1. Within +6hrs of C59 treatment *Grem1* transcripts (Fig. 3A) and proteins (Fig. S7A) are reduced to very low levels.

It would be worthwhile to explain the systems presented in figure 7 some more. With more components and interactions they become harder to understand at an intuitive level.
We agree that these Turing systems are non-intuitive. We have now made additions to the text, the figure legend, and also added a new supplementary figure to help explain the correspondences between the models proposed here, and classical 2-species Turing models. One of the most non-intuitive aspects of any Turing system, is that they either have a positive regulatory interaction between two components that are ***out-of-phase*** with each other (substrate-depletion model), or they have an inhibiting regulatory interaction between 2 components that are ***in-phase*** with each other. This is hard to explain in an intuitive way, but some attempts to do this can be found in Marcon et al. 2016, and Diego et al. 2018. We have graphically highlighted these non-intuitive elements in Fig. 7A,B.

For the substrate-depletion element (Fig 7D), comparing this to a simple two component substrate-depletion model there should be some freely-diffusing component that is present in limiting concentration and some other component that depletes it (as SULF1 does to WNT2), but the depleting factor should have limited diffusion and be self-enhancing? Which component(s) achieve these criteria or functions?
The reviewer is correct about the statement above. The factor which depletes the substrate (usually referred to as a self-activator), is a single molecule in the classical 2-species Turing system (act), and in our model this is implemented by a feedback loop between 4 molecules: Sox9 activates SULF1, which represses WNT2, which activates ß-Catenin, which represses Sox9. Because the feedback loop has an even number of repressions, this becomes a positive feedback loop, equivalent to self-activation. This is now illustrated in detail in Fig. S11A,B.

If WNT is the substrate then it must diffuse very readily and this is worth noting as a constraint, and then SULF1 achieves self-enhancement through suppressing WNT, which represses Sox9, which activates SULF1.
The self-enhancement loop (also described above) operates on the activator, not on the substrate (see Fig. S11A). So in fact WNT is part of the activator loop – it is not the substrate. This is therefore compatible with WNT having the more constrained diffusion. It should be noted however, that the popular view that Turing systems depend on differential diffusion is not true if the network has 3 or more nodes. This is true even if there are only two diffusible species (Diego et al. 2018).

For Fig 7B, the activator inhibitor system, however, WNT would seem to need to undergo more constrained diffusion, at least diffusing less than Sfrp2, if the latter is the inhibitor.

© 2025. Published by The Company of Biologists under the terms of the Creative Commons Attribution License (https://creativecommons.org/licenses/by/4.0/).

Yes, this is indeed correct. But as explained above more constrained diffusion of WNT is also compatible with the substrate/diffusion case – so in fact there is no contradiction or conflict between the 2 models - in both cases WNT may show the more constrained diffusion.

Then in 7E the degree to which this system should be regarded as activator-inhibitor or substrate-depletion could depend on the diffusivity of the WNT and how much WNT signal is blocked by SULF1 (S-D) compared to how much is blocked by Sfrp2 (A-I). BMP seems like it must diffuse readily in any of these models if it is to stimulate Sox9 expression in the digital mesenchyme.
Although we have now shown above that there is no contradiction in diffusion requirements for the two models, the reviewer is nevertheless correct that the full system may show more of the activator-inhibitor behaviour, or more of the substrate-depletion behaviour (or a balance of both) depending on the parameter values of the system.

I realise it is difficult to know where to stop with these explanations as they can become very wordy but a little more guidance for the reader on any key constraints or properties of the models, such as relative diffusion rates of species, and how these vary between the models presented, would be helpful.
On reflection we believe that the most valuable point to highlight is that indeed complex networks often do not require constraints on differential diffusion (as mentioned above).

Related to the models, are there really only two relevant expression domains (digit and interdigit) for the system? Fig 6D, for example, suggests that there might be more than this. The reviewer is correct that the spatial patterning become more complex than just 2 phases. A basic Turing model however can only explain the appearance of 2 phases. In this respect the modelling is therefore limited to explaining the appearance of the digital versus interdigital phases, and exploring whether the predicted expression of genes correctly correlates with these 2 domains. After the initial symmetry-breaking, certain genes, such as Sfrp2, are expressed in a distribution around the growing digit, and therefore seem to represent a 3rd phase. However, this cannot be captured by the model of initial symmetry-breaking.

In addition, Sulf1 appears to extend more distally than Sfrp2 - is Sulf1 expression activated earlier than Sfrp2's and would this be relevant to the model?
Again, unfortunately for the moment, the model can only deal with periodic symmetry breaking along the AP axis, and therefore the interesting temporal effects along the PD axis cannot be captured for now.
Furthermore RNA-FISH is not conclusive whether *Sulf1* expression is activated before Sfrp2. The earliest timepoint we detect *Sfrp2* and *Sulf1* in the distal limb bud is at E10.5. While the *Sfrp2* FISH signal is more distinct, *Sulf1* expression is not always detected or low. But this might simply be due to possibly different sensitivities of the HCR oligo probes and their amplification, which renders it impossible to conclude whether one gene is activated earlier or not. Therefore we refrain from discussing this in the manuscript.

In the text related to Fig 7 (p23) it is stated that SULF1 positively modulates BMP signalling, but in Fig 7E SULF1 has an inhibitory effect on BMP. Which is correct?
We thank the review for pointing out this error- the statement in the text was rather simplistic and misleading. The literature reports conflicting views dependent on the experimental approach used on the effects of SULF1 in modulating BMP signalling. Since this particular interaction is not required for the model, we have now removed it from the figure and text.

In Fig 7 legend specify what the grey versus black interaction lines signify
We now specify in the legend that the black arrows are those we have tested by computational modelling, while the grey arrows are hypothetical interactions that are compatible with the model.

In Fig S6B the Msx1expression at 6h looks stronger than in the control condition. Can the authors explain this?
This comment concerns the expression of *Msx2*: the rather homogenous signal in the core mesenchyme corresponds to non-specific fluorescence rather than reflecting generalized transcriptional up-regulation. We have repeated this HCR analysis and now show a forelimb bud with much less background.

© 2025. Published by The Company of Biologists under the terms of the Creative Commons Attribution License (https://creativecommons.org/licenses/by/4.0/).

Corrections:

p14 'constraint' --> 'constrained'
Done

p15 'wild type' would be better as 'control' or similar s genotypes are not different between conditions here
Agreed, as the wild-types have been IP injected with the DMSO carrier. Therefore, we now use the term wild-type and wildtype controls in text and figures.

p19 'WNT pathway is' --> 'as' Done

Fig S1 legend title has an extra '7' Done

Fig S2F should have a Sox9 label on the panel Done

The figures for Fig. S3 and Fig. S4 have been swapped and so sit above the wrong legend This has been corrected and former Fig. S4 is now Fig. S5.

Wnt2a is occasionally mentioned in the text but I think this is the same gene is more commonly referred to in the text as Wnt2. If there are three relevant Wnts (Wnt2, Wnt2a, Wnt2b) then please explain, if there are only two relevant Wnts (Wnt2=Wnt2a, Wnt2b) then please choose one designation and use that only throughout
This is correct and we have changed *Wnt2a* to *Wnt2* in text and figures.

REVIEWER 2

In this study, Malkmus et al. address the consequences of acute and transient Wnt signaling inhibition on mouse forelimb digit patterning, and identify the Wnt pathway at the core of a self-regulating Turing system. They employ the small molecule C59, which blocks the secretion of all WNT ligands, to transiently arrest Wnt signaling in embryos at 10.5 dpc during the early phases of forelimb bud development. Doing so allows expansion of the digit- specifying territory, marked by broadened expression of the cartilage master gene Sox9. Wnt signaling recovery at 36h post injection is sufficient to re-establish a self-regulatory system where digit and interdigit territories are marked, yet with delayed specification causing the reduction of digit number. The data foster a model whereby it is not only positional information driven by SHH - the most widely held view - but rather a complex network of feedback loops (similar to a Turing system) capable of self-regulating and resulting in repetitive digit-interdigit territories. The question underlying the study is important, and the methods employed to respond it elegant and persuasive.
We very much appreciate the positive evaluation, input and openness of this reviewer and have addressed all comments and suggestions in adding additional data and revising the text accordingly.

1) Three different inhibitors are compared and C59 is chosen based on the severity of phenotype. The study is therefore entirely based on using this compound. Formally, I would have expected that the choice of which inhibitor to use should be made based on a Wnt- specificity readout, for example by measuring which one leads to the strongest downregulation of Axin2, while basing this decision on the severity of phenotype induced might be seen as cherry-picking. Axin2 reduction is only shown for C59 (Fig. 1B). While the effects on gene regulatory networks measured in the rest of the study do point to a specific effect, I am left wondering why the other inhibitors did not cause any (or much) phenotype. Have the authors assessed Axin2 expression upon their injection? This would help to sort this out: the absence on effects on Axin2 would suggest that other inhibitors simply do not work in this context. If, on the other hand, IWP2 and ETC-59 caused Axin2 downregulation but no phenotype, this might indicate a possible toxicity/off-target consequences induced by C59, which would warrant additional controls.
As suggested by the reviewer, the initial screen included the analysis of *Axin2* expression by WISH in C59, IWP2 and ETC159 treated limb buds 6hrs after IP injection, which is now shown in Fig. S3A. While *Axin2* expression is not significantly reduced by 10µg/g IWP2, it is largely lost following injection of 10µg/g ETC-159, which causes digit phenotypes in a small fraction of forelimb buds

© 2025. Published by The Company of Biologists under the terms of the Creative Commons Attribution License (https://creativecommons.org/licenses/by/4.0/).

(Table in Fig S3B). Increasing the ETC-159 concentration to 20 µg/g causes loss of one digit (tetradactyly) in most forelimb buds (n= 10/11). These initial results showed that (1) IWP2 fails to rapidly and/or completely inhibit WNT signaling in mouse limb buds and that (2) the efficacy of ETC-159 in altering limb bud development is lower than the one of C59 (possibly due to differences in metabolic turnover). As C59 can be used at lower concentrations and had already been successfully used to inhibit WNT signaling during mouse embryonic and limb development (Szenker-Ravi et al. 2018, Nature 557, 564-569) and Axolotl limb regeneration (Lovely et al. 2022, Front. Cell Dev. Biol. 10:814250), we decided to use C59 for our study. These results are included in the revised Fig. S3 and described in more detail in the Figure legend to Fig. S3 and in the Result section (page 8). As pointed out by the reviewer, the downstream RNA-seq and gene network analysis (Fig.1, 2) is consistent with C59 blocking WNT signaling in mouse embryos following IP injection into pregnant mice. In addition, there is no widespread apoptosis as might be expected from drug toxicity, but very localised and transient apoptosis in the anterior mesenchyme that does not at all affect digit progenitor cells (Fig. S7F and Result section).

2) Throughout the study the authors looked at forelimbs, and do not show data on hindlimbs (apart from something that could be glimpsed in Fig. S4). As the latter are slightly delayed during development, this seems like the perfect chance of manipulating Wnt signaling in the two similar but not identical system in one go. If the authors have any image or data on the hindlimbs' phenotype across their experiments, I encourage them to include these in the study.
At the beginning of the project we assessed also the hindlimb morphologies and skeletal phenotypes that revealed the severe/complete disruption of digit development. These data are now included in the new Fig. S4 and briefly described on page 8 of the Results section. As hindlimb buds of E10.5 roughly correspond to forelimb buds at E10.0, the severe reduction/loss of digits is a possible consequence of altering the specification of the autopod/ digit progenitor populations (Palacio et al, 2025. Nat Commun 16, 1226), which precludes analyzing the transition from limb bud outgrowth and patterning to the periodic digit-interdigit pattern.

3) Sox9 expands into the sub-ectodermal mesenchyme upon beta-catenin deletion (S2D). What has made the authors exclude that, instead of an expansion of expression, sub- ectodermal and ectodermal cells are lost? After all, the surface ectoderm is an epithelium susceptible to disintegration without beta-catenin adhesive function, irrespectively of Wnt signaling activation.
This is independently supported by conditional inactivation in only the mesenchymal compartment using the Hoxa13Cre (Fig. S2F) and Prx1Cre transgenes (Hill et al., Dev Cell 8, 727ff) as well as by C59-mediated transient inhibition of WNT signaling. In all cases of Sox9 expression expands the distal mesenchyme is seen. Furthermore, no apoptosis is observed in the distal mesenchyme of C59-treated forelimb buds during expansion and restoration of Sox9 expression (Fig. S7F).

4) I truly appreciated the molecular data showing beta-catenin genomic occupancy by ChIP- seq, and how beta-catenin peaks vanish with the use of the inhibitor whilst H3K27ac remains unaffected. This is truly compelling. One comment on the signal profile in Figure 2B: the profiles of H3K27ac in control and C59 look to me identical (I cannot spot any pixel difference between the two). Normally, two ChIP-seq replicates of even the same experiment yield slight, even if minimal, differences. Could the authors make sure that no image was used twice by mistake? I also recommend showing the H3K27ac peaks (not only the signal profile): these would be nice to see in the main figure*, but if the authors do not like this option, they are also ok as supplementary dataset. *Unperturbed H3K27ac, in fact, while interesting per se as it indicates that chromatin conformation is not perturbed in these loci in the absence of beta- catenin, is also an important control that the experiment in C59 injected embryos worked, landing credibility that the disappearance of beta-catenin from these positions is specific and not a consequence of e.g. cell death.
We have carefully checked into this and indeed the H3K27ac heatmaps look similar but there is no mistake in e.g. using the same image twice. To be 100% sure we also generated a correlation plot, which shows that the genome-wide comparison of the wildtype and C59 treated H3K27ac profiles show a very high correlation but are not identical (0.98). The subtraction heatmap of DMSO and C59-treated H3K27ac ChIP profiles reveals the rather few differences between the two conditions (see Figure below). In revising the manuscript, we have replaced the panels in Fig. 2b with a version that better matches the color-range of the analysis provided on Github. We suspect that there was a conversion problem from the original Illustrator to a PDF file. As requested, we now include individual H3K27ac profiles for the relevant genomic landscapes in wildtype and C59-

© 2025. Published by The Company of Biologists under the terms of the Creative Commons Attribution License (https://creativecommons.org/licenses/by/4.0/).

treated limb buds (Figs S6, S8), which confirm the conclusions from global analysis. In addition, we have checked into apoptosis on the request of reviewer 3, which is very localized at the timepoint of RNAseq and ChIP analysis (+6hrs, Fig. S7F).

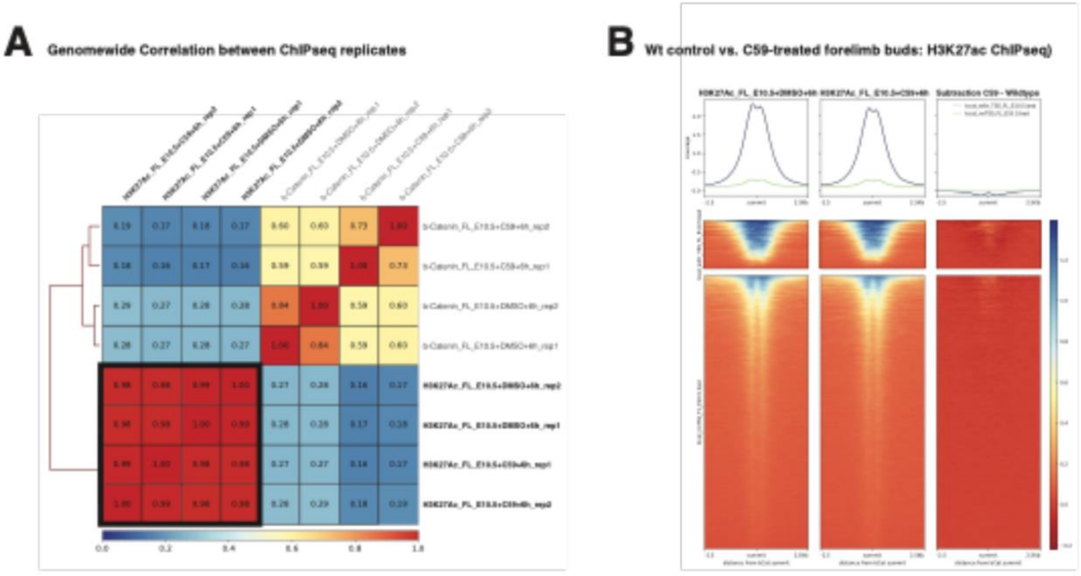

Note: Black frame indicates the relevant correlations between C59 and Wt replicates of the H3K27ac ChIPseq

Note: Subtraction reveals a small fraction of differences

5) Concerning the ChIP-seq on beta-catenin, the authors emphasize positively regulated direct targets. Yet their analysis point to considerable repressive action of Wnt signaling on the cartilage program. We have also seen a potentially direct ß-catenin-mediated downregulation, a phenomenon unexplained in the field (PMID: 37473729). Could the authors find beta-catenin-bound loci that match downregulated genes in their proximity? Interesting is for example the difference in behaviour between the adjacent Hoxd12 and Hoxd13.
In the revised manuscript, we include the ß-Catenin Chip Seq peaks in the genomic landscapes of *Sox9*, *Sulf1* and *Sfrp2*, which are upregulated in C59-treated forelimb buds (Fig. S8). In all three cases ß-Catenin peaks are detected in the region and in case of Sox9 at known active VISTA enhancer.
We agree that “the difference in behaviour between the adjacent Hoxd12 and Hoxd13” is very interesting. And indeed the ß-Catenin peaks are detected in the C- and T-Dom regions of the HoxD locus. However, the ChIP-seq analysis was done at E10.5 +6 hrs, at which 5’ HoxD gene expression is still posteriorly nested. However at E11.5 when we analyse *Hox* gene expression, their *cis*-regulation is very much altered due to distal-anterior expression (as studied in detail by the Duboule group. Therefore, the ß-Catenin peaks ChIPseq analysis done at E10.5 + 6hrs cannot be used to gain insight into *cis*-regulation of the late distal anteriorly expanded *HoxD* expression domains.

Minor points:
6) I find the use of the word "sensor" for target genes misleading. Reporter would be, in my opinion, better, as Axin2 is used as a proxy for the Wnt signaling status, while sensor implies the generation of an output based on the detection of a signal over a threshold.
We agree and have changed the few mentions of sensor to: (direct) transcriptional target *Axin2*.

7) At page 8, reference to Fig. S3 in describing the three inhibitors should be to Fig. S4 instead.
The Supplementary Figures (but not their legends) were inadvertently swapped in the Supplementary Figure File - this is now corrected. Note that former Fig. S4 is now Fig. S5.

8) In figure S2F the legend "Sox9" in the image is missing.
Corrected

9) At page 7, up in the paragraph there is a typo "during in": one of the two words should be deleted.

© 2025. Published by The Company of Biologists under the terms of the Creative Commons Attribution License (https://creativecommons.org/licenses/by/4.0/).

Corrected

10) At page 11, the reference to Fig. 2A on Grem1/Bmp peaks should be to Fig. 2G/2F.
Indeed the original text was somewhat ambiguous, therefore we have reworded it for clarity.

11) On page 14, "which indicates that the interdigit is a source for WNT ligand(s) functioning in digit-interdigit patterning (Fig. 4D)", perhaps the reference should be to 4E?
Corrected

12) In Figure 3A it is relatively straightforward to distinguish the three classic markers, for someone in the field, based on their position. Yet, it is not clear to me why the authors use a grayscale where one could not specifically distinguish Shh downregulation.
These three genes were detected by multiplexing in the same channel (Fig. 3A) to allow co-detect the FGF target *Dusp6* in the same limb bud (Fig. 3B). On the suggestion of reviewer 1 we now indicate the expression domains of the three genes in the first panel of Fig. 3A. In addition, we also include conventional WISH analysis in Fig. S7E to better illustrate the loss of *Shh* expression in C59-treated limb buds.

13) The authors suggest that Wnt targets might include Bmp2 (Figure 4B). We have previously found and focused on precisely this (see figure 11 of PMID: 30735122). Do the authors find the same peak within the Bmp2 locus in their ß-catenin ChIP-seq? If so, the results align and are a good support of each other.
We agree that there is good evidence for *Bmp2* being direct WNT target. Therefore, the *Bmp2* genomic landscape with all ß-Catenin and histone H3K27ac peaks is shown in Fig. S8B. Regarding the reviewer's specific question concerning the BCL9 ChIP-seq peaks reported in Salazar et al. (2019), we compared the peaks located at -152kb and -277kb (+/- 5kb) as well as the Bmp2 TSS with the ß-Catenin ChIPseq profile. This reveals a ß-Catenin peak at the region of the -152kb but not the -277kb BCL9 peak (**see Figure below**). In addition, there is a little peak at the TSS which not called. These data are not included as they is no complete correlation between the BCL9 and ß-Catenin peaks. More detailed analysis of potential BCL9/ß-Catenin interactions is beyond the scope of the present study.

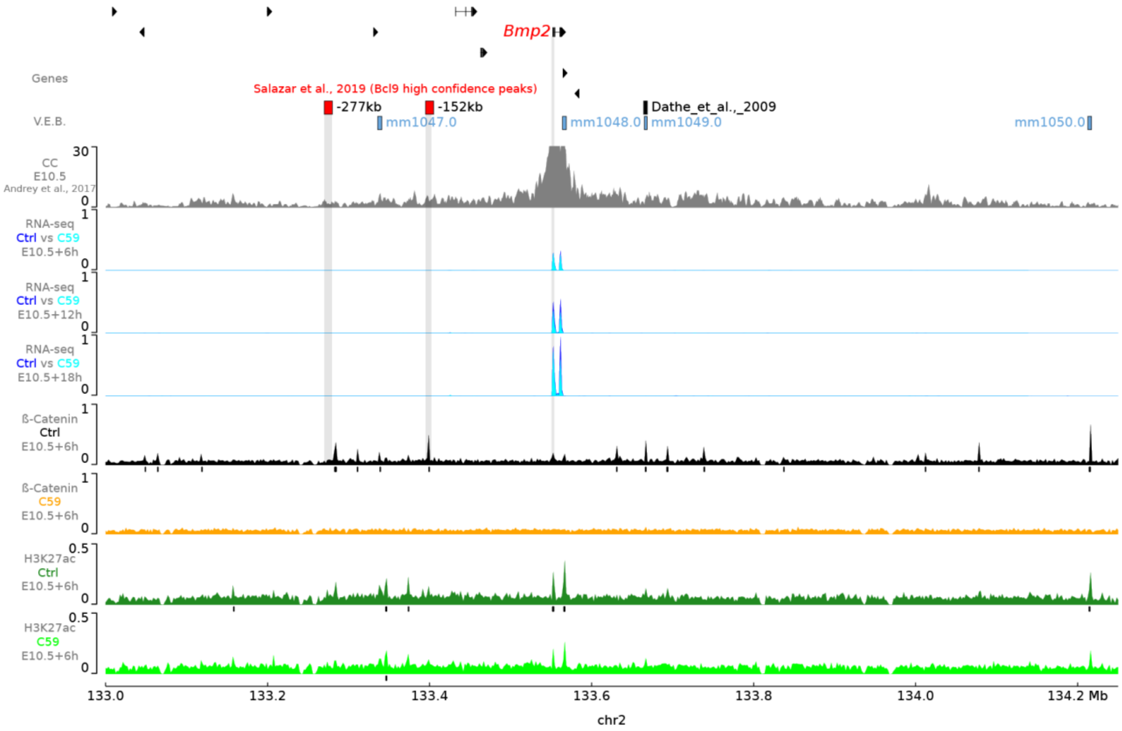

14) At page 6, the authors write "slow kinetics" and "disruptive nature" of genetic beta-catenin inactivation. I found these statement qualitative and weak as rationale to justify the use of C59. C59, in fact, inhibiting porcupine also has an arguably slow kinetics (compared to direct beta- catenin-

© 2025. Published by The Company of Biologists under the terms of the Creative Commons Attribution License (https://creativecommons.org/licenses/by/4.0/).

TCF/LEF inhibitors, for instance).
Agreed, we have rephrased the entire paragraph to explain the rationale for using transient inhibition rather than permanent genetic inactivation better (page 5).

Review by Claudio Cantù (Why I sign: I believe that peer review should be a transparent dialogue. I strive to be critical but honest and professional, and care that my opinions and criticisms are formulated as if I were meeting the authors in person).
We appreciate your openness!

Reviewer 3:

It was already known that WNT and BMP signaling establish early limb bud and digit-interdigit patterning, however, the molecular transition between these two processes remained unexplored. Here, Malkmus et al., propose WNT signaling as the controlling signaling pathway in this transition. Initially, by conditional inactivation of beta-catenin they showed that WNT/betacat signaling is required for digit-interdigit and phalange patterning. Nevertheless, this inactivation was permanent and did not allow to distinguish between early and late WNT functions. By injecting a WNT small molecule inhibitor, C59, they circumvent this problem and rapidly and transiently abrogate WNT signaling. The abrogation and later recovery of WNT signaling is nicely shown by FISH-RNA and RNAseq experiments. It is also shown how this reduction results in rapid and transient reduction of genes involved in SHH, FGF and BMP pathways and upregulation of chondrogenic markers. In order to assess whether this regulation is direct, beta-catenin and H3K27ac ChIPseq experiments were performed. The analysis showed that most of the described and/or potential enhancers of the DEG genes overlap with beta-cat binding regions. These bindings are absent upon C39 treatment. Therefore, it is concluded that WNT directly regulate these genes by interaction of beta-cat with their enhancers.
It is also shown that there is a disruption in the digit-interdigit patterning establishment in C59-treated limbs and Sfrp2 and Sulf1 are identified as candidates for WNT modulation during this process.
To clarify WNT function at later time points during digit ray progression, C39 was injected at E11.5. Again, alterations in gene expression involved in early limb bud signaling and digit-interdigit patterning were observed which are restored upon WNT recovery with no impact on digit development.
The restoration of digit patterning once WNT is reestablished, both in the early (10.5) and late (E11.5) C59-treated limbs, indicates the plasticity and robustness of this process.
Overall, I found the manuscript to be well-written, with clear results and providing molecular elucidation in digit-interdigit domains establishment.
We are also very thankful to this reviewer for the positive evaluation of our study and the helpful comments concerning our study. In particular, the proposed analysis of cell proliferation and apoptosis following C59 treatment is indeed an important aspect missing from the initially submitted study.

SUGGESTIONS TO AUTHORS

I have minor concerns about it. One of them is the lack of proliferation studies (more than just cluster C in figure 1D). Being WNT involved in the maintenance of limb mesoderm progenitors and given that the oligodactyly phenotype is caused by smaller handplates, a cell death/proliferation study is missing. Indeed, not just digits but all bones in stylopod and zeugopod are also smaller in the mutants (Fig.4D). Although this WNT role is known it would improve the study describing the dynamics of proliferation in C59-treated limb buds.
This is an excellent suggestion and the revised manuscript now includes the analysis of both cellular apoptosis and cell proliferation in Fig. S7F, 7G. Indeed, localized apoptosis is detected in the anterior (sub-ectodermal) mesenchyme at +6 and +12hrs in C59-treated forelimb buds, but no longer at +18 and +24hrs (Fig. S7F). In addition, we conducted comparative flowcytometric analysis of wildtype and C59-treated forelimb bud cells stained with DAPI for DNA content. This analysis (Fig. S7G) reveals the significant reduction of cells in S phase at +6hrs after C59-treatment. At +12 and +18hrs, cells in S and G2/M phase are decreased while cells in G1 are increased (Fig. S7G). This comparative analysis shows that LMP proliferation is reduced at 12 and 18hrs, but recovers at +24hrs after C59-treatment.

© 2025. Published by The Company of Biologists under the terms of the Creative Commons Attribution License (https://creativecommons.org/licenses/by/4.0/).

While it is shown that genes involved in digit pattering are affected, some of these genes are also present and affected in the stylopod and zeugopod regions (for instance Sox9 in figure 4A). Moreover, RNAseq is done with the whole limb. This makes difficult to conclude any digit specific result at least from RNAseq experiments (p.9 last paragraph : "...while inhibiting precocious chondrogenic fates and upregulation of genes function in digit patterning ").
We agree that this statement is premature and have removed it.

WNT non-canonical pathway has been shown to have a role in digit growth. How is the non-canonical pathway in your mutants? (RNAseq or FISH-RNA)
This is a good point, as C59 blocks WNT ligand secretion. It's important to note that non-canonical WNT signaling likely plays a role at later stages of limb bud development, since initial digit and interdigit patterning occurs correctly (Yamaguchi et al., 1999). As shown in Wnt5a mutants, non-canonical WNT signaling is involved in PFR formation (E11.75) and digit elongation via a convergent-extension mechanism (Parada et al., 2022; DOI: 10.1016/j.devcel.2022.03.004).
To address potential effect on non-canonical WNT signaling, we compiled gene lists based on canonical and non-canonical WNT GO terms, and included additional genes from general WNT signaling GO terms. Since many genes overlap, we created three groups for analysis: unique genes for canonical and non-canonical, and genes shared between the two. These lists were intersected with the DEGs identified in our forelimb bud RNAseq time course: this identified 23 DEGs associated to canonical (out of 443), 13 DEGs associated to shared (out of 91 genes) and no DEGs associated with genes of non-canonical WNT GO terms. As the GO-term based number of non-canonical WNT target genes is low, recently identified β- Catenin-independent WNT targets were added to the analysis (Voloshanenko et al., 2018; DOI: 10.1038/s41598-018-20641-5). This identified two DEGs (*Ndrg2*, *Ppp1r14c*) as potentially β-Catenin-independent targets, for neither of which there is functional evidence for an involvement in early limb bud development. Despite this significant data-mining effort, no significant support for a role of the non-canonical WNT pathway during early limb bud outgrowth and patterning, which is one focus of this study, was obtained.

I miss the skeletal phenotype of Ctnnb1betac/betac
The onset embryonic lethality of β-Catenin-deficient mouse embryos ≥E12.5-E13 precludes limb skeletal analysis-this is now stated in the manuscript text (page 7).

Figure legend of figure S3 and S4 are changed
This mistake has been corrected -the figures were swapped. Fig. S4 is now Fig. S5.

Figure S3 (s4?) Time course RNA-seq analysis. Section A is almost not explained and it is not clear to me what authors want to show. Remove?
This comment concerns what is now Fig. S5: we agree and have removed panel A. In addition we have relabelled the heatmap and its description in the Figure legend. We stated that this is linked to the 3 distinct clusters shown in Fig. 1D.

Minor:
p.7: during in (?)
Corrected
p.8 Fig. S3 A,B does not correspond
This is a consequence of the swap of Fig. S3 and S4 - all this has been corrected.
p.23 into results (?)
We concluded that the Turing simulations fit better the discussion of the potential network topologies underlying the periodic digit-interdigit pattern.
4E figure legend. Colocalization?? Do they colocalize? Same in Fig.5C,D
This reviewer is right, the better term in this context is co-detection (now changed).
Fig. S2F Sox9 labelling is missing
Corrected

Second decision letter

MS ID#: dev.204606R1

© 2025. Published by The Company of Biologists under the terms of the Creative Commons Attribution License (https://creativecommons.org/licenses/by/4.0/).

MS Title: WNT signaling coordinately controls mouse limb bud outgrowth and establishment of the digit-interdigit pattern

Authors: Jonas Malkmus; Angela Morabito; Lucille Lopez-Delisle; Laura Avino Esteban; Alexandre Mayran; Aimee Zuniga; James Sharpe; Rolf Zeller; Rushikesch Sheth
Article Type: Research Article

Dear Rolf

I am happy to tell you that your manuscript has been accepted for publication in Development, pending our standard publication integrity checks.

Reviewer 1

The manuscript is amended to take my comments into account

Reviewer 2

The authors have adequately responded to all my former criticisms and suggestions. Good luck!

Reviewer 3

Authors have responded clearly and satisfactorily to the recommendations I made. They have added the proliferation and cell death study which was the main suggestion.Therefore, I recommend the manuscript for publication , as it has demonstrated significant progress and meets the necessary standards

© 2025. Published by The Company of Biologists under the terms of the Creative Commons Attribution License (https://creativecommons.org/licenses/by/4.0/).
